# Tumor cell-activated “Sustainable ROS Generator” with homogeneous intratumoral distribution property for improved anti-tumor therapy

**DOI:** 10.7150/thno.50028

**Published:** 2021-01-01

**Authors:** Junjie Liu, Xiu Zhao, Weimin Nie, Yue Yang, Chengcheng Wu, Wei Liu, Kaixiang Zhang, Zhenzhong Zhang, Jinjin Shi

**Affiliations:** 1 School of Pharmaceutical Sciences, Zhengzhou University, Zhengzhou 450001, China.; 2 Key Laboratory of Targeting Therapy and Diagnosis for Critical Diseases, Henan Province, China, Zhengzhou University, Zhengzhou 450001, China.; 3 Collaborative Innovation Center of New Drug Research and Safety Evaluation, Henan Province, Zhengzhou, China, Zhengzhou University, Zhengzhou 450001, China.

**Keywords:** sustainable ROS generation, homogeneous tumor distribution, switched on, GSH depletion

## Abstract

Photodynamic therapy (PDT) holds a number of advantages for tumor therapy. However, its therapeutic efficiency is limited by non-sustainable reactive oxygen species (ROS) generation and heterogeneous distribution of photosensitizer (PS) in tumor. Herein, a “**S**ustainable **R**OS **G**enerator” (SRG) is developed for efficient antitumor therapy.

**Methods:** SRG was prepared by encapsulating small-sized Mn_3_O_4_-Ce6 nanoparticles (MC) into dendritic mesoporous silica nanoparticles (DMSNs) and then enveloped with hyaluronic acid (HA). Due to the high concentration of HAase in tumor tissue, the small-sized MC could be released from DMSNs and homogeneously distributed in whole tumor. Then, the released MC would be uptaken by tumor cells and degraded by high levels of intracellular glutathione (GSH), disrupting intracellular redox homeostasis. More importantly, the released Ce6 could efficiently generate singlet oxygen (^1^O_2_) under laser irradiation until the tissue oxygen was exhausted, and the manganese ion (Mn^2+^) generated by degraded MC would then convert the low toxic by-product (H_2_O_2_) of PDT to the most harmful ROS (·OH) for sustainable and recyclable ROS generation.

**Results:** MC could be homogeneously distributed in whole tumor and significantly reduced the level of intracellular GSH. At 2 h after PDT, obvious intracellular ROS production was still observed. Moreover, during oxygen recovery in tumor tissue, ·OH could be continuously produced, and the nanosystem could induce 82% of cell death comparing with 30% of cell death induced by free Ce6. For *in vivo* PDT, SRG achieved a complete inhibition on tumor growth.

**Conclusion:** Based on these findings, we conclude that the designed SRG could induce sustainable ROS generation, homogeneous intratumoral distribution and intracellular redox homeostasis disruption, presenting an efficient strategy for enhanced ROS-mediated anti-tumor therapy.

## Introduction

Reactive oxygen species (ROS) are highly important bio-active substances, mainly including superoxide anions (·O_2_^-^), hydrogen peroxide (H_2_O_2_), hydroxyl radicals (·OH) and singlet oxygen (^1^O_2_) 1, 2. Because of the unpaired electrons, ROS hold high chemical reactivity and can induce cell necrosis or apoptosis by inducing lipid peroxidation or damaging intracellular proteins and nucleic acids, which were considered as important therapeutic agents for tumor therapy 3, 4. Among a variety of ROS-mediated cancer treatments, photodynamic therapy (PDT) has attracted widespread attention due to its high selectivity and low side effects, which uses photosensitizers (PSs) to produce cytotoxic ROS under specific wavelengths of light, and has been approved for clinical treatment of a variety of solid tumors 5, 6.

The majority of clinic available PSs for PDT are mainly through Type II photodynamic processes, which convert oxygen into singlet oxygen through energy transfer 7-9. Therefore, its therapeutic effect is completely oxygen-dependent 10-12. However, most solid tumors are hypoxia and the PDT process will completely consume tissue free oxygen in a few minutes 9, 13, 14. When the oxygen is exhausted, PSs can no longer produce ROS even under light irradiation 15. Only when the oxygen is recovered, the^ 1^O_2_ can then be produced again. Therefore, the current PDT can hardly achieve sustainable and long-term treatment. Based on the O_2_-dependent nature of PDT, to date, various strategies have been proposed to relieve the hypoxic tumor microenvironment. For example, hemoglobin 15-17, perfluorohexane 18, 19 and other materials 13, 20-22 are used to transport oxygen directly to the tumor site or *in situ* catalyze H_2_O_2_ in tumor tissue to generate oxygen 23-28, which improves the efficiency of PDT for tumor treatment. However, such strategies are limited to the stability of oxygen-carrying processes and the content of H_2_O_2_ in the tumor 29-31, which is not enough to provide sufficient oxygen to sustain PDT. Even worse, the intermittent production of ROS always leads to the adaptive upregulation of reductase in tumor cells, which further reduces the therapeutic efficiency of PDT 32-34. Furthermore, during the process of PDT, the PS can also generate H_2_O_2_ and ·O_2_^-^ in addition to ^1^O_2_ 35, 36. And the overexpressed superoxide dismutase (SOD) in tumor cells further converts ·O_2_^-^ into H_2_O_2_ 37, 38. Because of the low toxicity of H_2_O_2_, the efficacy of PDT is further limited.

The efficient accumulation and retention of PSs in tumor tissue also determine *in vivo* PDT efficiency. Many previous studies have shown that nanoparticle-based delivery strategy can significantly improve the antitumor efficiency of PDT by improving the tumor distribution of PS owing to the classic enhanced permeability and retention (EPR) effect 39-42. Although the “nanostrategy” improves the efficiency of PDT for tumor treatment, the solid tumors always have a high degree of heterogeneity 43-45. Except for tumor cells, there are also many kinds of stromal cells, such as fibroblasts, macrophages, etc. in tumor tissue, and these cells also have typical location characteristics 46, 47. For example, fibroblasts and macrophages are generally located near the blood vessels, while tumor cells are relative far away from the blood vessels 48-50. Therefore, to enhance the therapeutic efficiency of PDT, it's necessary for PSs to accumulate in tumor tissue efficiently and simultaneously penetrate deep into tumor tissue. Moreover, tumor cells far from blood vessels show higher SOD and glutathione (GSH) expression, leading to a stronger antioxidant capacity 51, which will further limit the therapeutic effect of ROS-based treatment.

To address the above issues, herein, in this work, a “**S**ustainable **R**OS **G**enerator” (SRG) was designed for efficient antitumor therapy via homogeneous distribution in whole tumor, disruption of intracellular redox homeostasis and cascade ROS generation. The “sustainable ROS generator” was prepared via encapsulating small-sized Mn_3_O_4_-Ce6 nanoparticles into dendritic mesoporous silica nanoparticles (DMSNs) and then enveloped with hyaluronic acid (HA). SRG exerts its enhanced ROS-mediated antitumor effect depending on: **(I)** accumulation in tumor via EPR, while homogeneous distribution in whole tumor attributing to the significant particle size changes triggered by hyaluronidase (HAase), which is highly expressed in tumor stroma; **(II)** after endocytosis by tumor cells, the Mn_3_O_4_-Ce6 nanoparticles can rapidly disrupt intracellular redox homeostasis by converting the reduced glutathione (GSH) to oxidized glutathione (GSSH), simultaneous switching PDT on; **(III)** ^1^O_2_ is produced efficiently before the tissue oxygen is exhausted, and then Mn^2+^ produced by Mn_3_O_4_ nanoparticles can spontaneously convert the low toxic by-product (H_2_O_2_) of PDT into the most harmful ROS (·OH), triggering the cascade ROS generation, and achieving the continuous killing of tumor.

## Methods

### Chemicals and reagents

Chlorin e6 (Ce6) was obtained from meilunbio. N-(3-Dimethylaminopropyl)-N-ethylcarbodiimide hydrochloride crystalline (EDC), N-Hydroxysuccinimide (NHS) and Manganese acetate (MnC_4_H_6_O_4_·4H_2_O) were purchased from Aladdin. Hyaluronic acid sodium salt (3~10 kDa) was obtained from Bloomage Biotechnology Co., Ltd. HAase from bovine testes was purchased from Sigma. Hexadecyltrimethylammonium bromide (CTAB), Tetraethyl orthosilicate (TEOS) and (3-aminopropyl) triethoxysilane (APTES) were obtained from Sigma-Aldrich. DMSO was purchased from Tianjin HengXing Chemical Reagent co., LTD. GSH and GSSG Assay Kit was purchased from Beyotime Biotechnology. Hydroxyl radical (·OH) detection kit was obtained from BestBio. Mouse HAase ELISA Kit was purchased from Shanghai Lianshuo Biological Technology Co., Ltd.

### Characterization

Dynamic light scattering (DLS) was measured on the Zetasizer (Nano ZS-90, Malvern, UK). Transmission electron microscopy (TEM) was performed on a Tecnai G2 20 transmission electron microscope (FEI) with an acceleration voltage of 200 kV. Energy dispersive spectroscopy (EDS) mapping analysis was carried out on a GENESIS scanning electron microscope (EDAX). The UV-vis spectrum was characterized by a UV-vis spectrophotometer (UV-2550, Shimadzu). The fluorescence spectrum was measured with a RF-5301PC fluorescence spectrophotometer (Shimadzu).

### Synthesis of amino-modified manganese tetroxide nanoparticles (Mn_3_O_4_-NH_2_)

The Mn_3_O_4_-NH_2_ NPs were synthesized according to the previous literature with minor modifications 52. Mn (OAc)_2_·4H_2_O (2.45 g) was added to DMF (70 mL) and stirred until fully dissolved. Then the solution was transferred into an 80 mL Teflonlined stainless steel autoclave for thermal treatment for 8 h at 160 °C. After the solution cooled down, the products were transferred to a 100 mL round bottom flask and was heated to 130 °C, then APTES (500 µL) were added. The reaction solution was further stirred for 12 h and collected by centrifugation (12000 rpm, 5 min), washed with ethanol.

### Synthesis of manganese tetroxide -Ce6 conjugates (MC)

Ce6 (10 mg) were dissolved in DMSO (300 μL), EDC (3.2 mg) and NHS (2.3 mg) were dissolved in DMSO (100 µL), respectively. The EDC/NHS solution were added to the Ce6 solution in turn, and incubated at 37 °C for 20 min in dark. The activated Ce6 was added into Mn_3_O_4_-NH_2_ solution (25 mL, 200 mg) and stirred for 4 h. Free Ce6 was removed by centrifugation and washed with ethanol until the supernatant was colorless.

To evaluate the Ce6 loading on Mn_3_O_4_ NPs, the supernatant was collected and the absorption of unloaded Ce6 in the supernatant was detected by UV-vis absorption spectra. The concentration of Ce6 was calculated from its characteristic absorption peak at 400 nm by a standard curve of Ce6. The loading efficiency and encapsulation efficiency were calculated as follows: loading efficiency (%) = m_2_/m_0_ × 100%; encapsulation efficiency (%) = m_2_/m_1_ × 100%, where m_2_ is the weight of Ce6 in the Mn_3_O_4_ NPs, m_1_ is the weight of Ce6 added to the system, and m_0_ is the weight of Mn_3_O_4_ NPs.

### Synthesis of dendritic mesoporous silica nanoparticles (DMSNs)

DMSN was synthesized according to a previous reported protocol 53. Triethanolamine (68 mg) were dissolved in deionized water (25 mL), then, CTAB (380 mg) and sodium heptafluorobutyrate (74 mg) were added. The solution was stirred at room temperature. After addition of TEOS (4 mL), the solution was further stirred for 24 h. The products were centrifuged and followed by calcination at 550 °C for 6 h.

### Synthesis of amino-modified dendritic mesoporous silica nanoparticles (DMSN-NH_2_)

DMSN (200 mg) were dispersed into anhydrous toluene (20 mL). When the solution was heated to 116 °C, APTES (1 mL) was added under vigorous stirring. After refluxed for 24 h, the products were centrifuged at 5000 rpm for 5 min and washed with ethanol for three times, dried in vacuum overnight.

### Synthesis of SRG

DMSN-NH_2_ (4 mg) was dispersed in formamide (10 mL). After addition of MC (80 mg) under stirring, the solution was stirred at room temperature overnight. Meanwhile, HA (80 mg) were dissolved in formamide (10 mL), and then formamide solution (10 mL) containing EDC (138 mg) and NHS (82 mg) was added, the solution was stirred for 3 h. Then the MC@DMSN solution was added drop by drop into the above HA solution and further stirred for 24 h. After that, three times precooled acetone was added and the product was collected by centrifugation and washed with water.

### *In vitro* time-dependent degradation profiles of Mn_3_O_4_ NPs

Mn_3_O_4_ (at a final concentration of 200 μg mL^-1^) was mixed with 1 mM GSH for different time in water. The absorbance of the solution was recorded at the indicated time points by the UV-vis absorption spectra.

### *In vitro* GSH-dependent degradation profiles of Mn_3_O_4_ NPs

Mn_3_O_4_ (at a final concentration of 500 μg mL^-1^) was mixed with different concentrations of GSH for 30 min in water. The absorbance of the solution was recorded by the UV-vis absorption spectra.

### *In vitro* GSH-mediated Mn^2+^ release from MC

MC (1 mg mL^-1^) was pretreated with or without different concentrations of GSH for 30 min in water. The supernatant was collected by centrifugation at 12000 rpm for 20 min and Mn^2+^ content in the supernatant was measured by inductively coupled plasma mass spectrometry (ICP-MS).

### *In vitro* GSH-mediated Ce6 release from MC

MC (3 mg mL^-1^) was treated with different concentrations of GSH for 30 min at room temperature, and then the supernatant was collected by centrifugation at 12000 rpm for 20 min. The Ce6 fluorescence in the supernatant was measured by a fluorescence spectrophotometer.

### Mn_3_O_4_ mediated transformation of GSH to GSSG *in vitro*

The GSH and GSSG concentration was quantified by GSH and GSSG Assay Kit. Briefly, GSH (0.5 mM) was mixed with and without different concentrations of Mn_3_O_4_ (25, 50, 100 μg mL^-1^) for 30 min in water. Then, the solution was centrifuged at 12000 rpm for 10 min. The supernatant was carefully separated and analyzed according to the manufacturer's protocol.

### *In vitro* ^1^O_2_ generation ability

The ^1^O_2_ generation ability of MC was evaluated by singlet oxygen sensor green (SOSG). MC (Ce6: 1 μM; Mn_3_O_4_: 25 μg mL^-1^, pretreated with or without different concentrations of GSH for 30 min) was mixed with SOSG (4 μM) in water. The solution was then irradiated with a 660 nm laser at 0.2 W cm^-2^ for 2 min. After incubation for 30 min, the SOSG fluorescence with a 490 nm excitation and 525 nm emission was measured by a microplate reader.

### *In vitro* ·OH generation ability

The ability of Mn_3_O_4_ NPs to catalyze the production of ·OH in the presence of GSH was first confirmed by the degradation of methylene blue (MB). Briefly, NaHCO_3_ solution (25 mM) containing Mn_3_O_4_ (500 μg mL^-1^) was pretreated with different concentrations of GSH for 30 min. The supernatant was collected by centrifugation. Then MB (10 μg mL^-1^) and H_2_O_2_ (8 mM) was added, the solution was incubated at 37 °C for 30 min. The absorbance of MB at 400-800 nm was measured.

The ·OH generation ability of Mn_3_O_4_ NPs was also evaluated by the ESR measurement. NaHCO_3_ solution (25 mM) containing Mn_3_O_4_ (500 μg mL^-1^) was pretreated with GSH (1 mM) for 30 min. The supernatant was collected by centrifugation. DMPO was added into the solution and detected immediately after addition of H_2_O_2_ (8 mM), DMPO and Mn_3_O_4_ were used as controls.

### *In vitro* MR imaging and relaxivity calculation

SRG was incubated with 0.5 mg ml^-1^ HAase at 37 °C, and then the samples were treated with or without 10 mM GSH. *In vitro* MR images were acquired using a 3.0 T MRI system. The following parameters were adopted for data acquisition: (1) T_1_-weighted images (T_1_WI): echo time (TE) = 2.98 ms; repetition time (TR) = 100 ms; slice thickness = 2.0 mm; field of view (FOV) = 180 × 180 mm; (2) T_1_-Map images: TE = 2.57 ms; TR = 15 ms; slice thickness = 2.0 mm; FOV = 180 × 180 mm. Linear regression was performed between 1/T_1_ and [Mn] concentration, and the slope of the line obtained was the longitudinal relaxivity.

### Cellular culture

Murine breast cancer 4T1 cells and Human normal breast Hs578Bst cells were obtained from American Type Culture Collection (ATCC) and were cultured in RPMI-1640 medium containing 10% fetal bovine serum (FBS) and 1% penicillin/streptomycin at 37 °C under 5% CO_2_.

### Detection of intracellular GSH and GSSG

Hs578Bst cells and 4T1 cells (3×10^5^ per well) were seeded into a 6-well plate and cultured overnight. After treatment with different concentrations of MC, cells were digested by trypsin and collected by centrifugation. The sample was analyzed according to the manufacturer's protocol. The GSH level of Hs578Bst cells was evaluated by normalizing the measured values to the initial value of 4T1 cells.

### Cellular uptake

Hs578Bst cells and 4T1 cells (2×10^5^) were seeded in confocal dishes respectively and cultured overnight. Ce6 and MC (equivalent Ce6 concentration, 2 μM) were added and incubated for different time, respectively. Cells were then washed with PBS, fixed with 4% paraformaldehyde (15 min), stained with DAPI (15 min), and were observed by using the Leica confocal microscope (Germany).

### Intracellular H_2_O_2_ detection

4T1 cells (1.5×10^5^ per well) were seeded in a 12-well plate for 12 h. Ce6 (1 μM) was added and incubated for 4 h. a H_2_O_2_ indicator (BES-H_2_O_2_-Ac) dissolved in RPMI-1640 (at a final concentration of 50 μM) was incubated with 4T1 cells for 1 h, the cells were washed with RPMI-1640 medium and exposed to a 660 nm laser at a power of 0.2 Wcm^-2^ for 2 min. Then the sample was detected by a fluorescence microscope.

### Intracellular ROS detection

4T1 cells were seeded in 12-well plates and cultured overnight. After removing the culture medium, cells were incubated with Ce6 or MC (equivalent Ce6 concentration, 0.7 μM) for 4 h. After washed with PBS, the cells were exposed to a 660 nm laser at a power of 0.2 Wcm^-2^ for 2 min. After 0 h and 2 h, cells were incubated with DCFH-DA (10 μM) for 20 minutes. And then the fluorescence images were obtained by a fluorescence microscope. 4 h later, the cells were irradiated with a 660 nm laser at a power of 0.2 Wcm^-2^ for 2 min again, and incubated with DCFH-DA for 20 minutes. Subsequently, the fluorescence images were acquired using a fluorescence microscope.

### Detection of intracellular ·OH

The ·OH generation ability of MC inside cells was measured by the ·OH specific green fluorescent probe (BBoxiProbe^®^O26). 4T1 cells were seeded in 12-well plates and cultured overnight. After removing the culture medium, cells were incubated with Ce6 or MC (equivalent Ce6 concentration, 0.7 μM) for 4 h. After washed with PBS, the cells were exposed to a 660 nm laser at a power of 0.2 Wcm^-2^ for 2 min. BBoxiProbe^®^O26 was diluted 1000 times with serum-free medium. After 0 h and 2 h, cells were incubated with diluted BBoxiProbe^®^O26 for 1 h. And then the fluorescence images were obtained by a fluorescence microscope. 4 h later, the cells were irradiated with a 660 nm laser at a power of 0.2 Wcm^-2^ for 2 min again, and incubated with diluted BBoxiProbe^®^O26 for 1 h. Subsequently, the fluorescence images were acquired using a fluorescence microscope.

### Phototoxicity assay

The *in vitro* phototoxicity of varies preparations was investigated by CCK-8 assay. 4T1 cells (8×10^3^ per well) were seeded in a 96-well plate and cultured overnight. After removing the culture medium, cells were incubated with Ce6, MSN-Ce6 and MC for 4 h, respectively. Then the cells were washed with PBS and replaced with fresh culture medium. Subsequently, the cells were exposed to 660 nm irradiation (0.2 Wcm^-2^) for 2 min. After 24 h incubation, 10 μL CCK-8 was added into each well. After 3 h of incubation, the absorbance at 450 nm of each well was measured on a microplate reader.

### Cell apoptosis

4T1 cells (1.5×10^5^ per well) were seeded in a 6-well plate and cultured overnight. The medium was then replaced. Cells were incubated with Ce6, MSN-Ce6 and MC for 12 h and then exposed to 660 nm irradiation (0.2 Wcm^-2^, 2 min). The cells were further cultured for 12 h, then digested by trypsin, collected by centrifugation and washed with PBS. The cells were finally dispersed in binding buffer (0.8 mL), Annexin V-FITC (8 μL) and PI (8 μL) were added in turn. The samples were incubated at room temperature for 10 min, and then analyzed by flow cytometer.

### Detection of apoptosis-related proteins by western blot

To detect cell apoptosis, 4T1 cells were seeded in a 30-mm dish and cultured overnight. The cells were treated with Ce6, MSN-Ce6 and MC (equivalent Ce6 concentration, 2 μM) for 12 h, respectively, then the cells were replaced with fresh culture medium and exposed to 660 nm irradiation (0.2 Wcm^-2^, 2 min). Cells were further cultured for 12 h and collected by centrifugation. Cells were lysed on ice for 40 min in 100 μL lysis buffer and the lysates were collected by centrifugation (12000 rpm, 15 min). Protein concentrations were determined by BCA Protein Assay Kit. Total cellular proteins were separated by SDS-PAGE.

### *In vitro* penetration ability

To prepare the multicellular spheroids (MCSs), 4T1 cells (5×10^3^) suspended in medium (100 μL) were plated in a 96-well plate that was pre-coated with 1% low melting point agarose (100 μL). Seven days later, the uniform MCSs were selected and incubated with Ce6, DMSN-Ce6 and MC (the concentration of Ce6 was 2 μM) for 6 h and 12 h, respectively. Then, the MCSs were washed with PBS and transferred to confocal dishes. The Ce6 fluorescence in spheroids was measured with a confocal microscope.

### Animal models

Female Balb/c mice (15-18 g) were obtained from Henan Laboratory Animal Center and all the animal experiments were performed in accord with the guidelines of the Regional Ethics Committee for Animal Experiments and Zhengzhou University Institutional Animal Care and Use Committee. To develop the tumor model, 4T1 cells (2×10^6^) suspended in RMPI-1640 medium (200 µL) were subcutaneously injected on the underarm of the mice.

### *In vivo* HAase detection

Four 4T1 tumor-bearing mice were randomly selected. The liver and tumor tissue were excised and washed with PBS. The weighed liver and tumor tissue of each group were cut into small pieces, and the precooled PBS buffer containing 1% protease inhibitor was added, the tissues were then homogenized thoroughly on ice. The homogenized liquid was transferred to the centrifuge tube, and centrifuged at 5000 g for 5 min at 4 °C. The supernatant solution was carefully separated and analyzed by the Mouse HAase ELISA Kit.

### *In vivo* blood circulation and tissue distribution

The mice were intravenously injected with SRG (Mn_3_O_4_: 10 mg kg^-1^). 10 μL blood was collected from the tail vein at 15 min, 30 min, 1, 2, 4, 8, 12 and 24 h (n = 5 at each time point), respectively. The samples were digested with HNO_3_ for 1 h and Mn^2+^ concentrations were detected by ICP-MS.

To evaluate the tissue distribution, the 4T1 tumor-bearing mice were sacrificed at 12 h after intravenous injection of SRG (Mn_3_O_4_: 10 mg kg^-1^). Major organs and tissues (the heart, liver, spleen, lung, kidney, tumor, stomach, intestine and skin) were collected, weighted and lysed with HNO_3_ and H_2_O_2_ mixture solution. Mn^2+^ concentrations in various organs were measured by ICP-MS.

### *In vivo* imaging

4T1 tumor-bearing mice were depilated and intravenously injected with Ce6, MC and SRG (equivalent Ce6 concentration, 2.0 mg kg^-1^ body weight), respectively. Fluorescence imaging was performed at designed times with a 670 nm excitation and 790 nm emission. At 24 h post-injection, the heart, liver, spleen, lungs, kidneys and tumor were excised for *ex vivo* imaging.

MR imaging was also performed in the tumor tissue after injection. 4T1tumor-bearing mice were intravenously injected with SRG (200 µL, 200 µg mL^-1^). After 2, 4, 6 and 8 h, MR imaging was captured under a 7.0 T magnetic field for small animal imaging.

### *In vivo* deep penetration

4T1 tumor-bearing mice were intravenously injected with Ce6, MC@DMSN@F-68 and SRG (equivalent Ce6 concentration, 5.0 mg kg^-1^ body weight), respectively. At 24 h post-injection, the mice were sacrificed and the tumors were collected for frozen section.

### Hypoxia immunofluorescence staining of tissue

Pimonidazole can be reductively activated in hypoxic cells and forms stable adducts with thiol (sulphydryl) groups in proteins, peptides and amino acids. Then FITC-MAb1 binds to these adducts allowing tissue staining of hypoxia. Briefly, 4T1 tumor-bearing mice were intravenously (i.v.) injected with SRG (equivalent Ce6 concentration, 5.0 mg kg^-1^ body weight). At 6 h post-injection, the tumor was irradiated with a 660 nm laser at a power density of 0.2 Wcm^-2^ for 5 min. At 0, 3, 6 and 12 h post-irradiation, the mice were intraperitoneally injected with pimonidazole HCl (60 mg kg^-1^). After that, the mice were sacrificed and the tumors were collected. Frozen tumor sections were treated with FITC-MAb1 antibody and HRP conjugated rabbit anti-FITC secondary antibody following the kit's instructions. Nucleus was stained with DAPI (blue) and hypoxia areas were stained with antipimonidazole antibody (green). Images were obtained by microscopy.

### *In vivo* antitumor efficacy

4T1 tumor-bearing mice were divided into four groups (five mice in each group): (1) Saline; (2) Ce6 + Laser; (3) DC + Laser; (4) SRG + Laser. The mice were injected with different preparations (equivalent Ce6 concentration, 5.0 mg kg^-1^ body weight) through the tail vein. At 6 h post-injection, the tumor was irradiated with a 660 nm laser at a power density of 0.2 Wcm^-2^ for 5 min. After different treatments, tumor size and body weight of the mice were measured every 2 d for 2 weeks. The tumor volume was calculated according to the following formula: length×width^2^×0.5. The relative volume of tumors was evaluated by normalizing the measured values to their initial sizes. On the 14th day of treatment, the mice were sacrificed and the tumors were photographed and weighed to evaluate the anti-tumor effect. The major organs (heart, liver, spleen, lungs, and kidneys) and the tumor were sectioned for H&E staining. To further assess the therapeutic effect, tumors were also sectioned and stained for ROS and apoptosis detection.

### Statistical analysis

All the data were from at least three independent measurements (n ≥ 3). All data were presented as mean ± standard deviation (SD). Statistical analysis was conducted with OriginPro (version 7.5) via Student's T-test and one-way analysis of variance (ANOVA) at confidence levels of 95% and 99%, respectively.* P* values <0.05 were considered significant (*P<0.05, **P<0.01 and ***P<0.001). As for *in vivo* photodynamic studies, mice were assigned randomly to treatment groups.

## Results and Discussion

### Construction and characterization of SRG

**Scheme 1:**
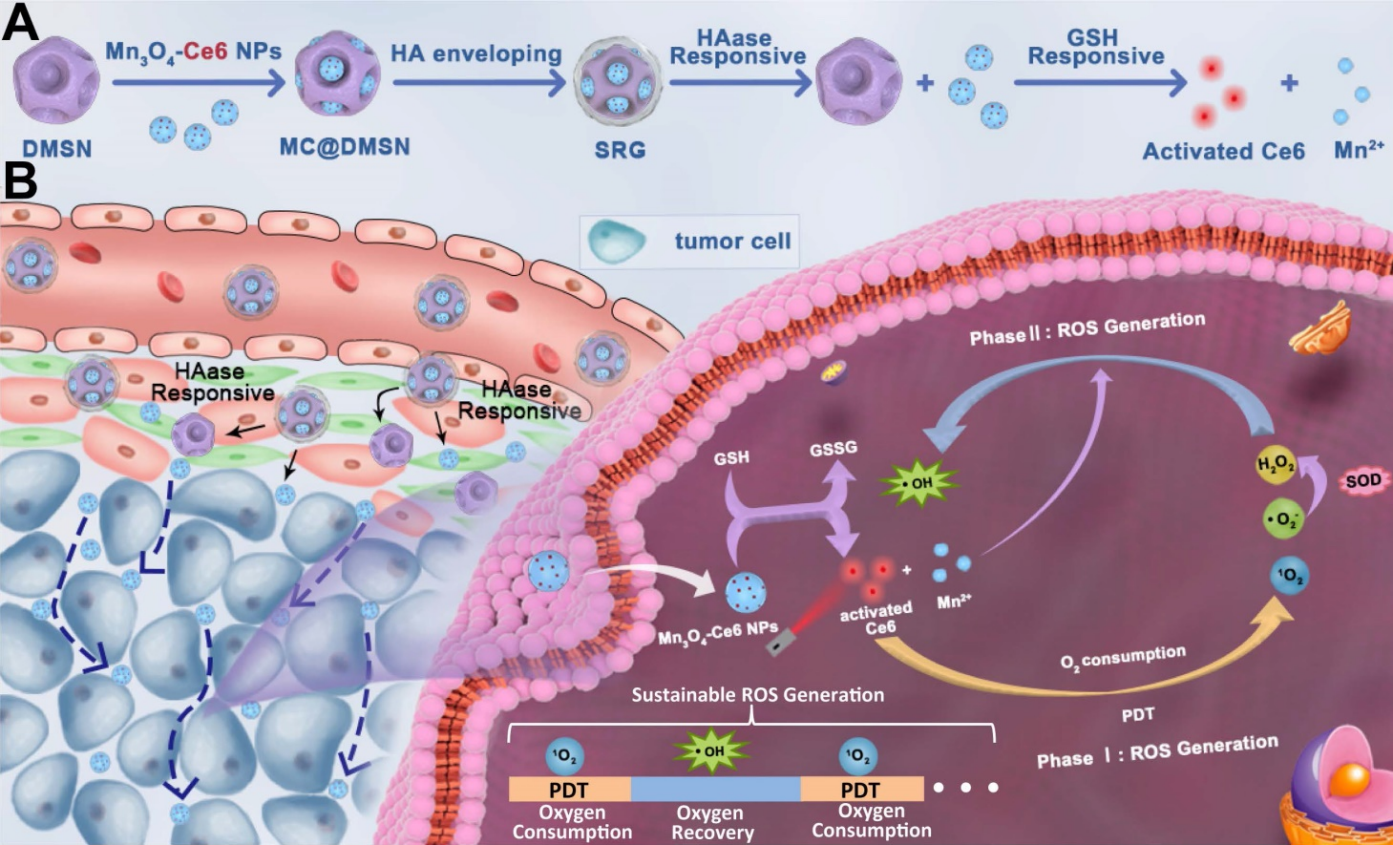
** Illustration of** (**A**) Preparation of the SRG, HAase and GSH-induced sequential response release of MC and Ce6. (**B**) *In vivo* behavior of SRG: tumor accumulation, deep penetration, tumor cell-specific switch on of PDT and sustainable ROS generation.

**Figure 1:**
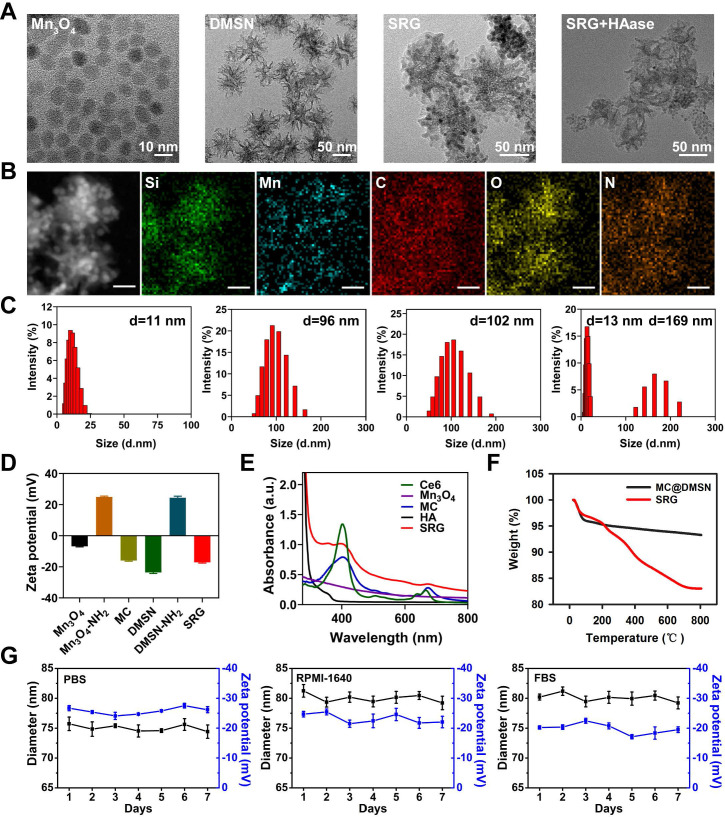
** Characterization of SRG.** (**A**) TEM images of different preparations. (**B**) STEM-EDS elemental mapping images of SRG (Scale bar: 25 nm). (**C**) The dynamic light scattering images corresponding to the TEM images of (**A**). (**D**) Zeta-potential of varies preparations (n=3). (**E**) UV-vis spectra of varies preparations. (**F**) TGA curves of MC@DMSN and SRG. (**G**) SRG was incubated in PBS, RPMI-1640 and FBS at 37 °C, respectively, the diameter and zeta potential were recorded as a function of time.

The detailed synthetic procedure of the “Sustainable ROS Generator” (SRG) is illustrated in **Scheme 1A**. To construct SRG, Mn_3_O_4_ NPs were first prepared via a hydrothermal method. Transmission electron microscopic (TEM) showed that the as-synthesized Mn_3_O_4_ nanoparticles (Mn_3_O_4_ NPs) with spherical structure were well dispersed in DMF and its average diameter was ~8 nm (**Figure 1A**), which was also verified by dynamic light scattering (DLS) measurements (~11 nm) (**Figure 1C**). The successful synthesis of Mn_3_O_4_ NPs was further confirmed by the X-ray diffraction (XRD) pattern (**Figure S1**). X-ray photoelectron spectroscopy (XPS) was conducted to confirm the chemical states of elements. As shown in **Figure S2**, the survey spectrum in which carbon (C), oxygen (O) and manganese (Mn) elements were in their respective oxidation states. And the spin-orbit splitting peaks were observed at 641.4 and 653.2 eV, which were assigned to Mn 2p_3/2_ and Mn 2p_1/2_, respectively. To formulate Mn_3_O_4_ NPs for biomedical PDT applications, Mn_3_O_4_ NPs were treated with (3-aminopropyl) triethoxysilane (APTES) to modify amino groups, and the PS, chlorin e6 (Ce6), was conjugated onto the surface of Mn_3_O_4_ NPs via an amide reaction 54. After Ce6 conjugation, the zeta potential of Mn_3_O_4_ NPs was reversed from a positive value of 24.9 mV for Mn_3_O_4_-NH_2_ to a negative one of -16 mV for Mn_3_O_4_-Ce6 (MC) (**Figure 1D**). And the UV absorption spectrum of MC showed the characteristic absorption peaks of Ce6 at 400 and 660 nm (**Figure 1E**), which confirmed the successful modification of Ce6 on Mn_3_O_4_ NPs. The Ce6 loading efficiency was ca. 4.2 wt% and the encapsulation efficiency was ca. 83%. Then, dendritic mesoporous silica nanoparticles (DMSNs) were prepared at room temperature. As shown in **Figure 1A**, the average diameter of DMSNs was ~60 nm and there were large dendritic pores (~20 nm) on its surface 53. The average hydrodynamic diameter of the DMSNs was ~96 nm (**Figure 1C**), and its composition was identified by the X-ray diffraction (XRD) pattern (**Figure S3**). Next, the synthetic small MC (size: ~8 nm) were loaded into the large dendritic pores (~20 nm) of DMSNs, and then HA, as the gatekeeper, was covalently grafted onto the surfaces of DMSNs by an amide reaction, the final product Mn_3_O_4_-Ce6@DMSN@HA (SRG) was obtained. As revealed by TEM (**Figure 1A**), MC were evenly encapsulated into DMSNs. The homogeneous distributions of Si, Mn, C, O and N in SRG were confirmed by the EDS-elemental mapping (**Figure 1B**), further indicating that MC were successfully loaded into DMSNs. Both the characteristic peaks of Mn_3_O_4_ NPs and DMSN were observed in the X-ray diffraction (XRD) pattern of MC@DMSN and SRG (**Figure S4**), further proving the successful synthesis of MC@DMSN and SRG. When MC@DMSN was treated with GSH, the diffraction peaks of Mn_3_O_4_ NPs disappeared, suggesting that Mn_3_O_4_ NPs were degraded. However, after GSH treatment, the characteristic peaks of SRG had no obvious change. The possible reason was that HA protected Mn_3_O_4_ NPs from GSH degradation. Next, the functionalization of MC@DMSN with HA was confirmed by UV-vis and thermogravimetric analysis (TGA), respectively. As showed in **Figure 1E**, there was the characteristic absorption peak of HA in the UV absorption spectrum of SRG. TGA curves of MC@DMSN and SRG were shown in **Figure 1F**, regarding to SRG, an additional weight loss of 10.25% could be ascribed to the removal of the HA polymer on its surface. The stability of SRG was further studied, as reflected in **Figure 1G,** there was no observable change in the hydrodynamic diameter or zeta potential of SRG in one week, indicating that SRG was stable in PBS, RPMI-1640 and FBS, laying the foundation for *in vivo* applications.

To investigate the HAase-responsive release of MC, morphological changes of SRG before and after HAase treatment were investigated by TEM (**Figure 1A**), after treated with HAase, MC were released from DMSNs and distributed around DMSNs. The changes in the particle size of SRG were also measured by DLS (**Figure 1C**), there were two kinds of particles with size of ~13 nm and ~169 nm in the aqueous solution of SRG after treated with HAase, indicating that HA shell was degraded by HAase and the small MC were released from DMSNs. The large particle size of DMSNs might be due to its easy aggregation without the modification of HA.

**Figure 2:**
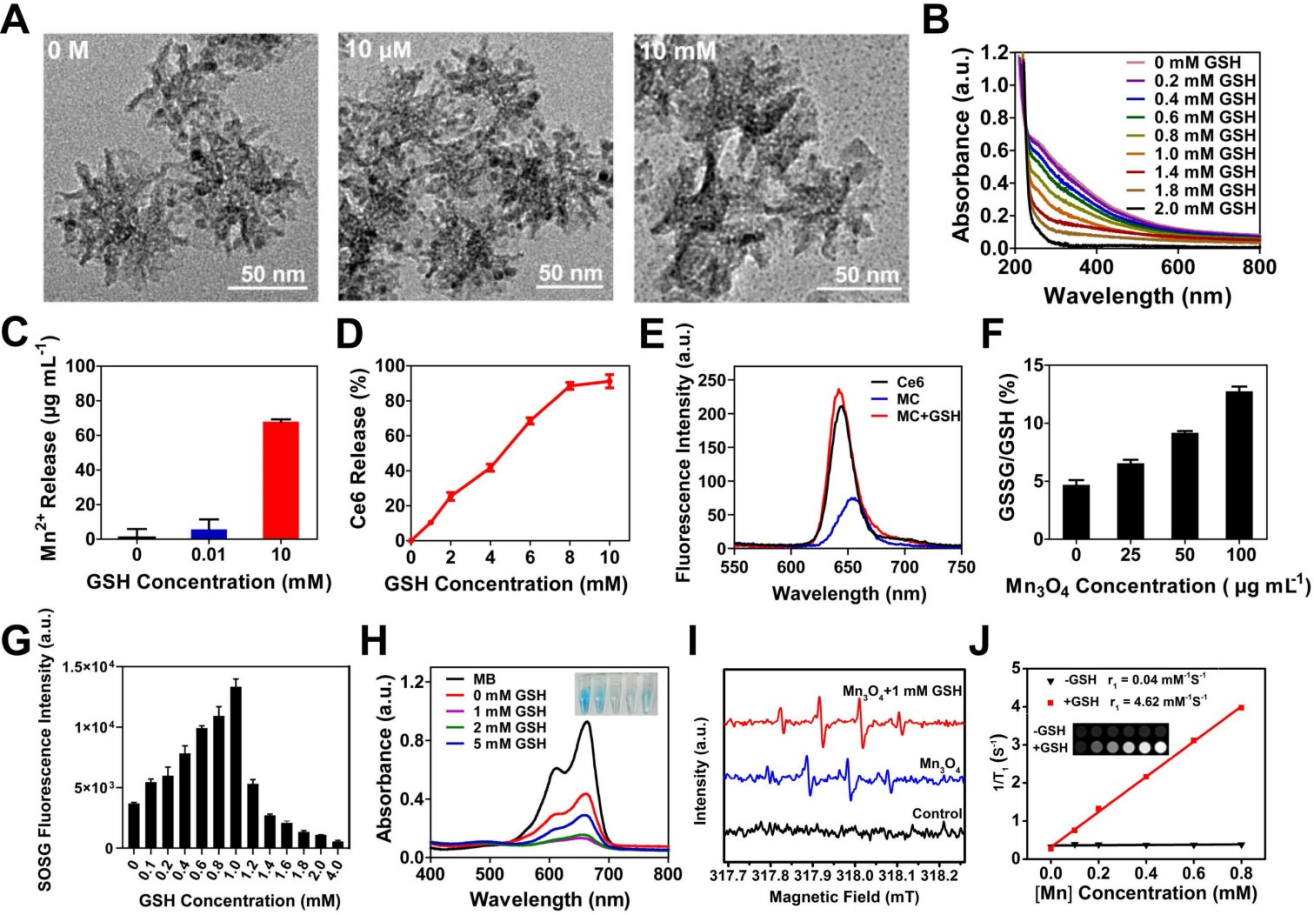
** GSH-activatable generation of ^1^O_2_ and ·OH.** (**A**) TEM images of MC@DMSN treated with 0 M, 10 μM and 10 mM GSH for 30 min. (**B**) UV-vis absorption spectra of Mn_3_O_4_ treated with different concentrations of GSH. (**C**) The Mn^2+^ release of MC after treated with different concentrations of GSH. (**D**) GSH-dependent release profiles of Ce6 from MC as measured by the fluorescence spectrophotometer. (**E**) Fluorescence intensity of Ce6, MC and MC treated with 1 mM GSH. (**F**) The transformation of GSH to GSSG after treated with different concentrations of Mn_3_O_4_. (**G**) Response of ^1^O_2_ generated by MC to different concentrations of GSH. (**H**) UV-vis absorption spectra and photo (inset) of MB after degraded by Mn_3_O_4_ NPs treated with different concentrations of GSH. The concentration of H_2_O_2_ is 8 mM. (**I**) ESR spectra of different reaction systems with DMPO as the spin trap. (**J**) T_1_ relaxivity of SRG pretreated with 0.5 mg ml^-1^ HAase in the absence and presence of 10 mM GSH. The [Mn] concentration was 0, 0.1, 0.2, 0.4, 0.6 and 0.8 mM. The insets were corresponding T_1_-weighted images.

Mn_3_O_4_ NPs have optical absorption at 200-800 nm, which makes them quench the fluorescence of other substances 25. Most importantly, there was a redox reaction between GSH and Mn_3_O_4_ NPs, leading to the decomposition of Mn_3_O_4_ NPs and the fluorescence recovery. Thus, Mn_3_O_4_ NPs can be used as a drug carrier to realize selective PDT. First, the degradation of Mn_3_O_4_ NPs by GSH was investigated by the UV-vis absorption spectra. The UV-vis absorption spectra suggested that Mn_3_O_4_ NPs were almost completely degraded at 30 min (**Figure S5**). Moreover, with the increase of GSH concentration from 0 M to 2 mM, the absorbance of Mn_3_O_4_ NPs decreased gradually, further proving that Mn_3_O_4_ NPs were decomposed by GSH and exhibited GSH-dependent degradation (**Figure 2B**)**.** Next, the degradation of Mn_3_O_4_ from MC@DMSN and SRG by GSH were studied by TEM. As displayed in** Figure 2A**, the morphology of MC@DMSN did not change in the presence of 10 μM GSH (simulate the tumor microenvironment). However, Mn_3_O_4_ NPs were completely degraded when treated with 10 mM GSH (simulate the tumor cell environment), indicating the GSH-dependent Mn_3_O_4_ degradation. The degradation of Mn_3_O_4_ from MC@DMSN by GSH also showed a time-dependent manner (**Figure S6**). However, Mn_3_O_4_ NPs in SRG couldn't be degraded and the morphology of SRG remained intact under the action of 10 mM GSH for 30 min (**Figure S7**). This might be because HA protected Mn_3_O_4_ NPs from GSH degradation. Then, the GSH-responsive release of Mn^2+^ was explored. As shown in **Figure 2C**, much Mn^2+^ was released from MC when treated with 10 mM GSH; however, negligible Mn^2+^ release was observed when treated with 10 μM GSH, indicating the GSH-dependent Mn^2+^ release. The release of Mn^2+^ from MC@DMSN and SRG in the presence of GSH was also investigated. The Mn^2+^ release from MC@DMSN showed concentration-dependent (**Figure S8**) and time-dependent (**Figure S9**). And the negligible Mn^2+^ was released from SRG in the presence of 10 mM GSH (**Figure S8**). Moreover, with the increased GSH concentration, Ce6 was gradually released from MC and ca. 92% of the Ce6 was released in the presence of 10 mM GSH (**Figure 2D**). Meanwhile, the fluorescence of Ce6 was restored by the addition of GSH (**Figure 2E**), and had a GSH concentration-dependent manner (**Figure S10**). Tumor cells have higher levels of GSH than normal cells, indicating that MC could be used for tumor-selective PDT.

Subsequently, the transformation product of GSH was detected (**Figure 2F**). Mn_3_O_4_ could effectively convert GSH to GSSG in a concentration-dependent manner, laying the foundation for intracellular GSH consumption. The conversion of GSH to GSSG can reduce the consumption of ROS by GSH and improve the efficiency of PDT 25, 55-61. Next, the GSH-responsive generation of ^1^O_2_ from MC were evaluated using a singlet oxygen sensor green (SOSG) reagent (**Figure 2G**). There was little singlet oxygen (^1^O_2_) generation without GSH treatment, and the production of ^1^O_2_ increased first and then decreased with the increased GSH concentration, indicating that ^1^O_2_ generation could be activated by GSH, while excess GSH would consume ^1^O_2_. As demonstrated in many previous studies, Mn^2+^ can catalyze the production of ·OH from H_2_O_2_ in the presence of HCO_3_^-^ 62-65**.** Therefore, Mn^2+^ released from MC can convert the low toxic H_2_O_2_ of PDT into the most harmful ·OH, triggering the continuous production of ROS, and achieving the continuous killing of tumor. The ability of Mn^2+^ to catalyze the production of ·OH in the presence of HCO_3_^-^ was first confirmed by the degradation of methylene blue (MB). The absorbance of MB decreased and the color became lighter when incubated with H_2_O_2_ and MnCl_2_ for 30 min at 37 °C (**Figure S11**). Next, the generation of ·OH catalyzed by GSH-treated Mn_3_O_4_ NPs was also investigated. As reflected in **Figure 2H**, the weakening of MB color and the decrease of absorbance was observed in the presence of GSH, attributing to that the GSH triggered Mn^2+^ release from Mn_3_O_4_ NPs, then inducing ·OH production in the presence of H_2_O_2_. However, due to the consumption of ·OH by excess GSH, MB degradation was suppressed when GSH was greater than 1 mM. The ·OH generation was also investigated by the electron spin resonance (ESR) spectroscopy (**Figure 2I**), 5, 5-dimethyl-l-pyrroline-N-oxide (DMPO) was used as a radical capture agent. After adding H_2_O_2_, the strong ESR signals in GSH-treated Mn_3_O_4_ group demonstrated the ·OH generation, while there was no ·OH generation in control group. In summary, these results showed that Mn_3_O_4_ NPs treated with GSH could catalyze the production of ·OH in the presence of H_2_O_2_. While the produced ·OH by Mn_3_O_4_ NPs may be attributed to the residual Mn^2+^ on the surface of Mn_3_O_4_ NPs. The released Mn^2+^ could also be used for MR imaging. Next, the T_1_-field MRI signal of SRG was examined. As shown in **Figure 2J,** SRG treated with HAase and GSH exhibited a paramagnetic property with an r_1_ value of 4.62 mM^-1^ s^-1^ and the T_1_-field MRI signal gradually increased with the increased SRG concentration. However, SRG treated with HAase (without GSH) had a weak T_1_-field MRI signal and the r_1_ value was 0.04 mM^-1^ s^-1^, indicating that SRG could be used for *in vivo* MR imaging.

### GSH depletion and tumor cell activation characteristics of MC

**Figure 3:**
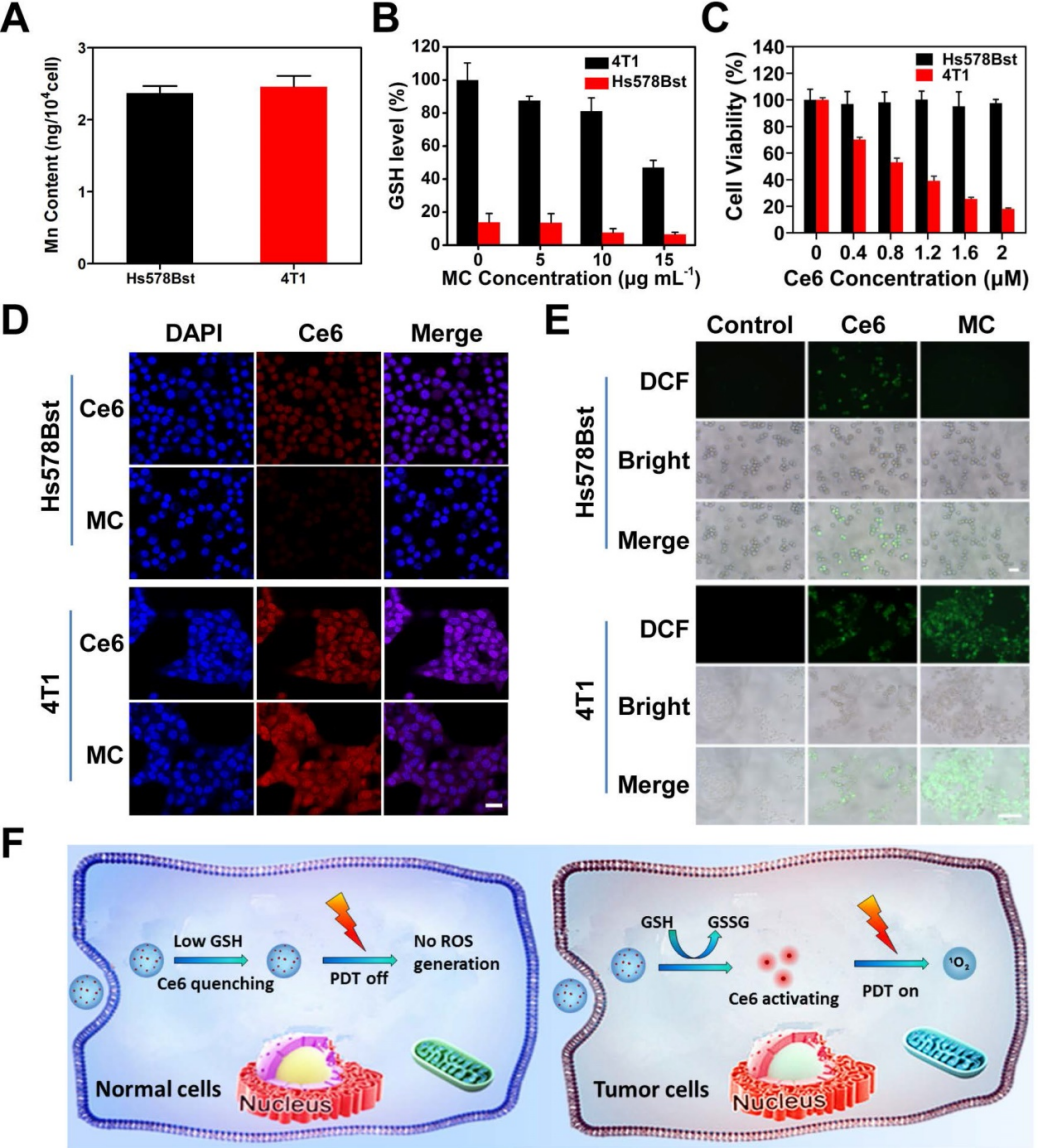
** Activation of MC in tumor cells.** (**A**) Mn content in cells, Hs578Bst cells were treated with MC for 6 h and 4T1 cells were treated with MC for 4 h. (**B**) The GSH level of different cells after treatment with different concentrations of MC, respectively. (**C**) Phototoxicity of MC to Hs578Bst cells and 4T1 cells after 660 nm irradiation for 2 min. (**D**) CLSM images of different cells incubated with Ce6 and MC, respectively (scale bar: 25 µm). (**E**) Fluorescence imaging of ROS production in different cells after different treatments (scale bars: 200 µm). (**F**) Schematic illustration of specific switched on of PDT in tumor cells.

The selectivity of MC toward tumor cells and normal cells was studied (**Figure 3F**). The 4T1 murine breast cancer cells with high expression of GSH were chosen as tumor cells, and human normal breast Hs578Bst cells with low level of GSH were chosen as control cells. To ensure the same uptake of MC by normal cells and tumor cells, Mn content in cells was measured by inductively coupled plasma mass spectrometry (ICP-MS) (**Figure 3A**), when Hs578Bst cells was incubated with MC for 6 h and 4T1 cells was incubated with MC for 4 h, the uptake of MC was the same. So, the uptake time of 6 h for Hs578Bst and 4 h for 4T1 cells was used for the subsequent experiments. We first investigated the effect of MC on GSH level in Hs578Bst cells and 4T1 cells. As shown in **Figure 3B**, GSH content in 4T1 cells decreased gradually with the increased MC concentration, however, MC had little effect on GSH content in Hs578Bst cells due to the low GSH basal value in normal cells, confirming that MC could reduce the GSH level in 4T1 cells and break intracellular redox homeostasis. The tumor cell-selective release of Ce6 was also examined by the confocal laser scanning microscopy (CLSM). As shown in **Figure 3D**, after Ce6 treatment, 4T1 cells and Hs578Bst cells both exhibited red fluorescence (Ce6). 4T1 cells treated with MC showed significantly high fluorescence intensity, however, only a weak fluorescence signal was observed in Hs578Bst cells. The obvious difference in fluorescence intensities suggested selective release of Ce6 from MC in tumor cells. In addition, 4T1 cells treated with MC presented stronger fluorescence than that of treated with free Ce6, suggesting that MC could deliver Ce6 effectively to tumor cells. Next, intracellular ROS generation was investigated by the fluorescence microscope (**Figure 3E**). 4T1 cells treated with MC showed strong DCF fluorescence, indicating the Ce6 release and ROS generation. However, there was no DCF fluorescence in Hs578Bst cells, confirming that Ce6 was deactivated by Mn_3_O_4_ NPs and ROS could not be produced. Free Ce6 was not affected by the quenching effect of Mn_3_O_4_ NPs, and ROS could be produced in both cells. Cell viability assay (**Figure 3C**) further proved that MC exhibited no obvious phototoxicity against Hs578Bst cells, while showed great killing effect on 4T1 cells and displayed a concentration dependent manner. Taken together, these results demonstrate that the high selectivity of MC toward tumor cells.

### Intracellular sustainable ROS generation

**Figure 4:**
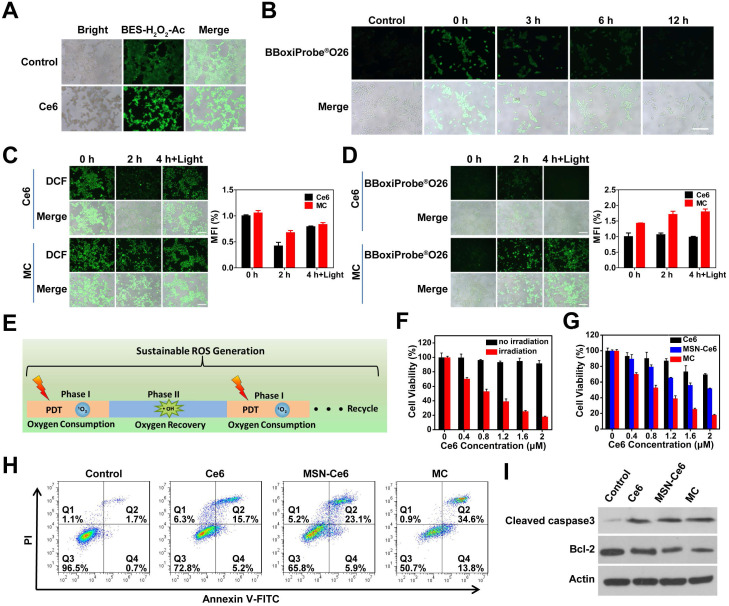
** Sustainable ROS generation in 4T1 cells.** (**A**) Fluorescence imaging of H_2_O_2_ production in 4T1 cells after different treatments (scale bar: 200 µm). (**B**) Fluorescence imaging of ·OH production at different time points after 4T1 cells (pretreated with 200 µM H_2_O_2_) incubated with 20 µg mL^-1^ Mn_3_O_4_ NPs for 4 h (scale bar: 200 µm). (**C**) Fluorescence imaging of ROS production in 4T1 cells at 0, 2 and 4 h after 660 nm irradiation (scale bars: 200 µm) and the corresponding mean fluorescence intensity (%) (n=3). (**D**) Fluorescence imaging of ·OH production in 4T1 cells at 0, 2 and 4 h after 660 nm irradiation (scale bars: 200 µm) and the corresponding mean fluorescence intensity (%) (n=3). (**E**) Schematic illustration of sustainable ROS generation of MC in tumor cells. (**F**) Cytotoxicity of MC in the presence or absence of irradiation. (**G**) Phototoxicity of Ce6, MSN-Ce6 and MC with Ce6 at different concentrations. (**H**) Flow cytometer analysis of 4T1 cells apoptosis following treatment with different preparations. (**I**) Western blot analysis with antibodies against cleaved caspase-3, bcl-2 and actin.

Subsequently, the feasibility of sustainable ROS generation for MC was assessed by the fluorescence imaging. The H_2_O_2_ level during PDT in 4T1 cells was first evaluated by a H_2_O_2_ indicator (BES-H_2_O_2_-Ac), which shows high selectivity for H_2_O_2_ and can generate green fluorescence. As shown in **Figure 4A**, cells treated with Ce6 had stronger green fluorescence, indicating that H_2_O_2_ was produced during PDT. The quantitative analysis result (**Figure S12**) was also consistent with this. And then the duration of ·OH generation through the Mn^2+^-mediated Fenton-like reaction was assessed (**Figure 4B**). At 0 h post-incubation, a large number of ·OH were produced. Although the produced ·OH gradually decreased with the extension of time, ·OH production was still observed at 6 h post-incubation, indicating that the production of ·OH by the Mn^2+^-mediated Fenton-like reaction could last for 6 h. The lasting ·OH generation was the prerequisite for sustained ROS production. Next, the ROS production in 4T1 cells was explored by a ROS probe (DCFH-DA) and the fluorescence intensity of DCF in 4T1 cells was measured. As shown in **Figure 4C**, after laser irradiation, a large amount of ROS was generated in 4T1 cells after treated with Ce6 and MC. At 2 h post-irradiation, the production of ROS declined slightly in cells treated with MC, however, the production of ROS in cells treated with Ce6 declined significantly. To clarify the mechanism, the content of ·OH in 4T1 cells was measured. BBoxiProbe^®^O26 (the ·OH specific green fluorescent probe) can be oxidized by ·OH in cells to produce green fluorescent products. As displayed in **Figure 4D**, there was strong green fluorescence in the MC-treated cells. However, no fluorescence signal was observed in Ce6-treated cells, which was consistent with the fluorescence quantitative results, indicating that Mn^2+^ produced by Mn_3_O_4_ NPs could spontaneously convert H_2_O_2_ into ·OH. After 4 h, when the oxygen was recovered, the cells were given a second laser irradiation, Ce6 and MC-treated cells showed strong DCF fluorescence (ROS). However, only MC-treated cells showed a strong fluorescence signal of ·OH, indicating that cells treated with MC could realize the sustainable ROS generation (**Figure 4E**).

### *In vitro* cytotoxicity of SRG

Mn_3_O_4_ NPs showed low cytotoxicity when its concentration was lower than 70 µg mL^-1^ (**Figure S13**). To identify the phototoxicity of MC, cell viability assay was conducted. As shown in **Figure 4F**, with the increased MC concentration, cell viability gradually decreased under laser irradiation, however, MC had low cytotoxicity without irradiation. To prove the advantage of MC in PDT, mesoporous silica nanoparticles (MSNs) were chosen as a control. Cell viability assay (**Figure 4G**) showed that all three formulations of Ce6 exhibited a concentration-dependent cytotoxicity against 4T1 cells under laser irradiation. When the concentration of Ce6 was 2 μM, Ce6 and MSN-Ce6 had 30% and 48% inhibition on 4T1 cells, respectively. While MC had 82% inhibition on 4T1 cells, which might be due to the synergistic effects of GSH depletion and sustainable ROS generation. Moreover, the intracellular ROS production was measured by flow cytometer (**Figure S14**), 4T1 cells treated with MC had higher fluorescence intensity than those treated with Ce6 or MSN-Ce6. And MC had the strongest capability to induce 48.4% apoptosis of 4T1 cells (**Figure 4H**), consistent with the cell viability assay result. Next, the expression of pro-apoptotic and anti-apoptotic proteins was evaluated using western blot. As shown in **Figure 4I**, compared with the control group, the pro-apoptotic protein (cleaved caspase-3) was increased and the anti-apoptotic protein (bcl-2) was decreased. More importantly, compared with Ce6 and MSN-Ce6 groups, MC group exhibited the highest expression of cleaved caspase-3 and the lowest expression of bcl-2, further confirming MC could effectively induce apoptosis. These results indicated that the GSH depletion and sustainable ROS generation induced by MC could improve antitumor efficiency of PDT. Finally, HA modification of particles was proved to be beneficial to its intracellular uptake. As shown in **Figure S15,** 4T1 cells treated with Ce6@DMSN@HA presented stronger fluorescence than that of treated with Ce6@DMSN@F-68, suggesting that particles modified with HA had a higher cellular uptake via the HA receptor medicated endocytosis pathway.

### Homogeneous distribution in 3D MCSs

**Figure 5:**
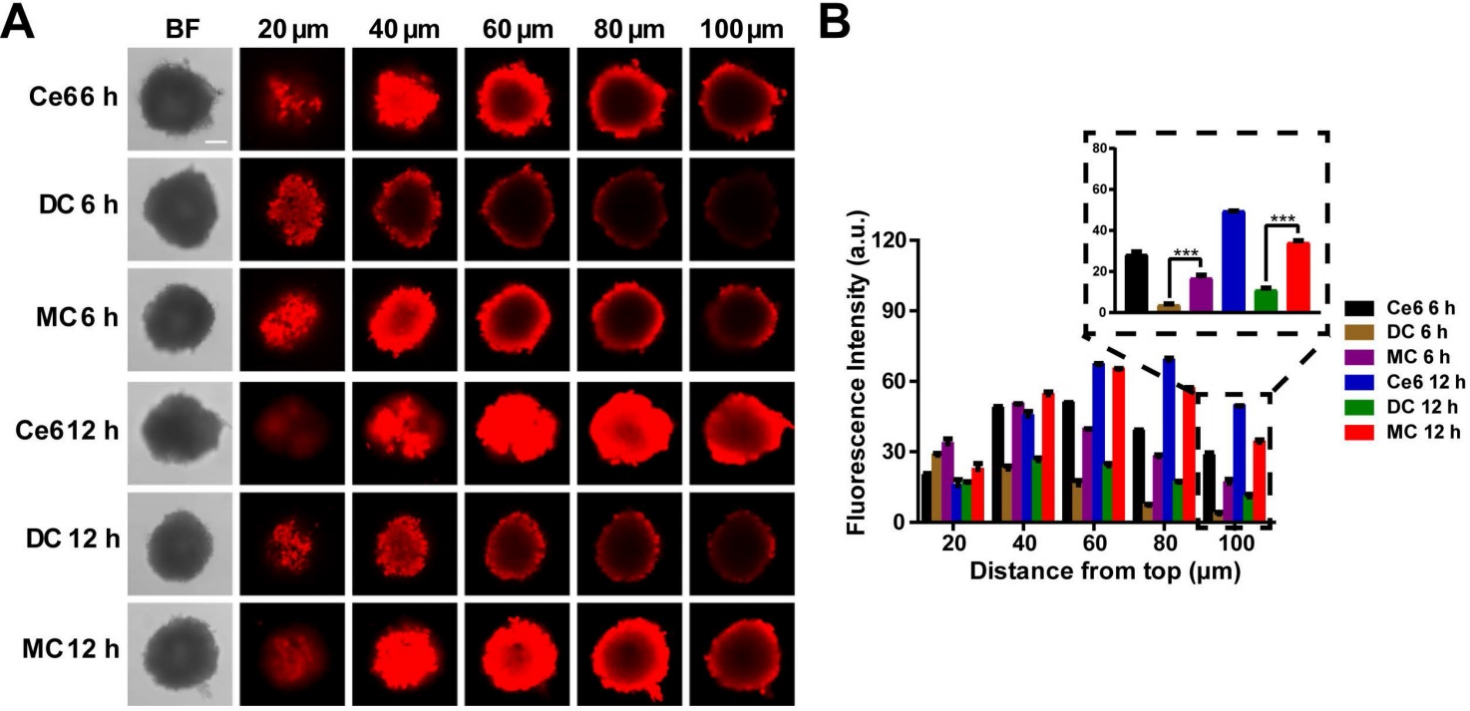
***In vitro* 3D MCSs penetration.** (**A**) Fluorescence imaging of the MCSs at different depths after treatment with Ce6, DC and MC for 6 h and 12 h, respectively (scale bar: 100 µm). (**B**) Fluorescence intensity of the MCSs at different depths after treatment with Ce6, DC and MC for 6 h and 12 h, respectively. Results are presented as means ± s.d. *P < 0.05, **P < 0.01, and ***P < 0.001 determined by Student's t test.

Next, the three-dimensional multicellular spheroids (3D MCSs) derived from 4T1 breast cancer cells were chosen as an *in vitro* model. Large-sized DMSNs loaded with Ce6 (DC) were prepared via covalent bond. As shown in **Figure 5A**, relatively large DMSNs (~50 nm) were mainly located in the periphery of the cell sphere and exhibited low penetration, whereas small-sized Mn_3_O_4_ NPs (~8 nm) could penetrate the core of the spheroids as well as free Ce6. All the three groups exhibited the time-dependent penetration. Moreover, according to the fluorescence quantitative analysis (**Figure 5B**), at 6 h and 12 h, the Ce6 fluorescence intensity of MC group was 4.6-fold (*p* < 0.001) and 3.1-fold (*p* < 0.001) that of DC group at 100 μm depth of 3D MCSs, confirming that small nanoparticles were more likely to penetrate into the central region of the 3D tumor spheroid.

### *In vivo* behavior of SRG

**Figure 6:**
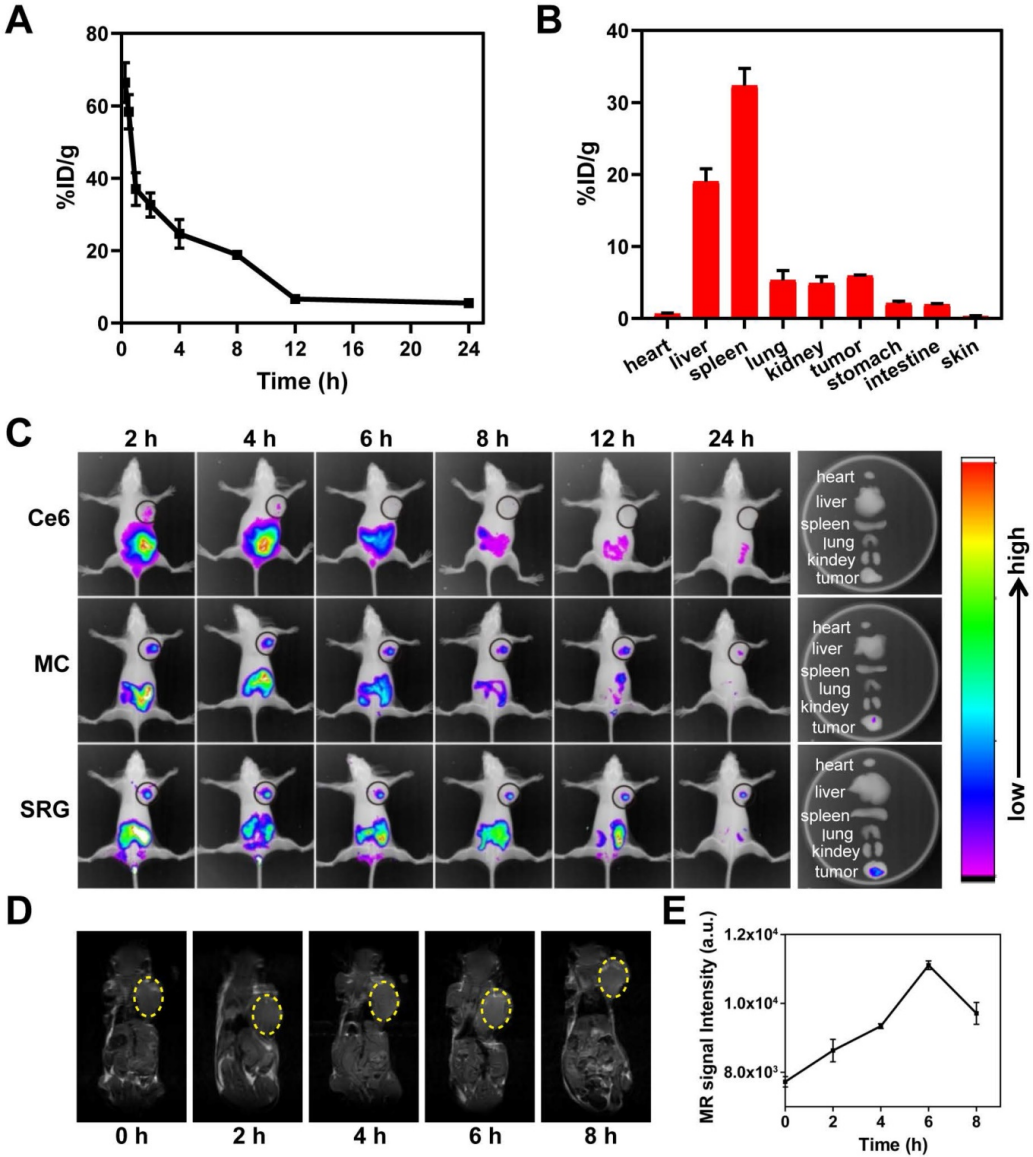
***In vivo* imaging and behaviors of SRG.** (**A**) Blood circulation curve of SRG by measuring the Mn^2+^ concentration in blood at different time points post-injection. (**B**) Biodistribution of SRG in mice at 12 h post-injection. The concentration of Mn^2+^ was measured by ICP-MS. (**C**) *In vivo* fluorescence intensity of tumor-bearing mice after intravenous injection with Ce6, MC and SRG, respectively and the corresponding fluorescence intensity of tumor and major organs at 24 h post-injection. (**D**) T1-weighted images of mice injected intravenously with SRG. The yellow circles indicate the tumor region. (**E**) Variation in the MR signals intensity in (**B**).

The blood circulation of SRG was studied by measuring the Mn^2+^ concentration in blood. As showed in **Figure 6A**, the level of SRG in the blood gradually decreased over time, which was in accordance with a two-compartment model by secondary exponential fitting. The first (t_1/2(_α_)_) and second (t_1/2(_β_)_) phases of circulation half-lives was calculated to be 0.35 ± 0.02 h and 6.69 ±0.05 h, respectively. The long blood circulation time was beneficial for SRG to enrichment in tumor tissue through EPR effect. Next, the biodistribution of SRG was investigated. In addition to a large accumulation of SRG in metabolic organs including the liver and spleen, significant accumulation of SRG was also observed in the tumor tissue (**Figure 6B**), indicating that SRG could efficiently accumulate in tumor tissues via the EPR effect. Moreover, the HA modification was beneficial to the accumulation of SRG in tumor tissues, the Mn^2+^ content in tumor tissues of tumor-bearing mice treated with SRG was 1.6-fold of mice treated with MC@DMSN@F-68 (**Figure S16**). Next, the *in vivo* distribution of Ce6, MC and SRG were investigated in 4T1 tumor-bearing mice. As showed in **Figure 6C**, free Ce6 group had a weak fluorescence signal in the tumor site at 2 h post-injection, and then the fluorescence decreased rapidly. On the contrary, a strong fluorescence signal was detected in the mice treated with MC and SRG, suggesting that MC and SRG could deliver Ce6 to tumor tissue efficiently via the EPR effect. Moreover, for SRG group, there was still strong fluorescence at 24 h post-injection, while the fluorescence at the tumor site almost disappeared in MC-treated mice, confirming that large-sized SRG had more accumulation in the tumor than small-sized MC. *Ex vivo* imaging at 24 h post-injection was also conducted. Highest fluorescence in the tumor of mice treated with SRG was observed compared to other treatments. In addition, MRI signals were also measured in the tumor tissue after injection and there was a maximum MRI signal at 6 h post-injection (**Figure 6D**-**E**), demonstrating that SRG could be used as an MRI contrast agent owing to the Mn^2+^ produced by Mn_3_O_4_ NPs.

**Figure 7:**
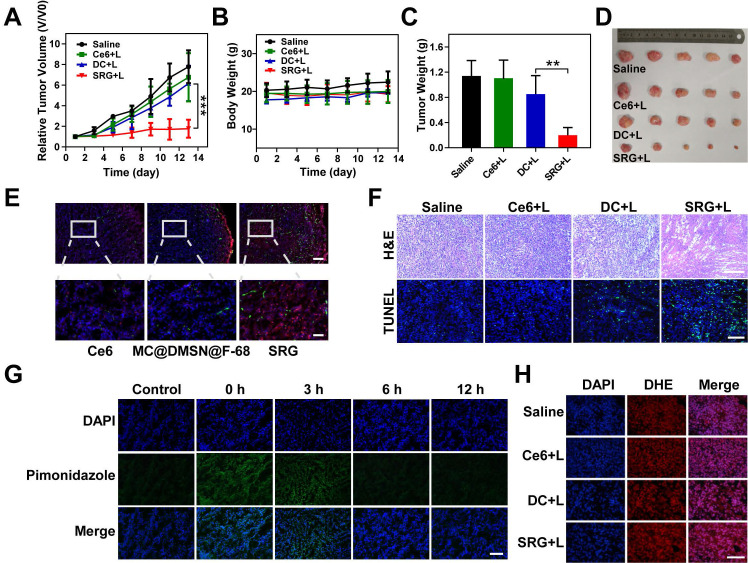
***In vivo* PDT of SRG.** (**A**) Tumor volume curve of mice upon different treatments (five mice for each group), Light irradiation (L) was conducted with a 660 nm laser at the power density of 0.2 Wcm^-2^ for 5 min. (**B**) Body weight curve of mice upon different treatments. (**C**) Weight of tumors collected from mice on the 14th day. (**D**) Representative photos of the *ex vivo* tumor. (**E**) Immunofluorescence staining images of frozen tumor sections. Tumor blood vessels were labeled with anti-CD31 antibody (green). Ce6 is shown in red and the nucleus in blue (DAPI) (scale bar: 100 µm). The following pictures were the enlarged parts among corresponding pictures (scale bar: 25 µm). (**F**) H&E and TUNEL staining of tumor tissues from different groups; scale bar (H&E): 200 µm; scale bar (TUNEL): 100 µm. (**G**) Immunofluorescence staining images of frozen tumor sections at different time points after PDT. The nucleus was stained with DAPI (blue), and hypoxia areas were stained with antipimonidazole antibody (green) (scale bar: 100 µm). (H) Fluorescence images of ROS production in tumor tissue. ROS is shown in red (DHE) and the nucleus in blue (DAPI) (scale bar: 100 µm). Results are presented as means ± s.d. *P < 0.05, **P < 0.01, and ***P < 0.001 determined by Student's t test.

Following the promising tumor accumulation of SRG, whether SRG could penetrate deep into tumor tissue was further explored. The expression of HAase in tumor and normal liver tissues were first investigated (**Figure S17**), the HAase content in tumor tissue was about twice that of liver tissue, indicating the high expression of HAase in tumor tissue, which was the basis for release of small-sized MC in tumor microenvironment. MC@DMSN@F-68 was prepared as a control, which couldn't release MC in tumor microenvironment. As showed in **Figure 7E**, for Mn_3_O_4_-Ce6@DMSN@F-68 treatment, no fluorescence signal appeared far away from the tumor blood vessels, suggesting its low penetration into the tumor tissue. In contrast, Ce6 fluorescence appeared far away from tumor blood vessels and was distributed in overall tumor tissues after SRG treatment, confirming that small Mn_3_O_4_ NPs had better penetration ability to make the homogeneous Ce6 distribution in tumor tissue.

Next, we assessed the time required for oxygen recovery in the tumor tissue by hypoxia immunofluorescence staining. As demonstrated in **Figure 7G**, compared to the tumors of untreated mice, the pimonidazole-stained (green) hypoxic signals were greatly enhanced after PDT, suggesting that PDT consumed a lot of oxygen, which leaded to tumor hypoxia. Subsequently, oxygen recovered gradually and recovered almost completely at 6 h after PDT. In summary, according to the above results, ·OH could be continuously produced for 6 h, earning 6 h for oxygen recovery of tumor tissue, which provided the possibility for continuous ROS production.

### *In vivo* antitumor efficacy

Based on the above results, the therapeutic efficacy of SRG was further estimated *in vivo*. The mice were irradiated with a 660 nm laser at the power density of 0.2 Wcm^-2^ (**Figure S18**). As shown in **Figure 7A**, compared to the saline group and Ce6 group, DC showed a slight tumor suppressive effect, and SRG had a complete inhibition on tumor growth due to the high tumor penetration and sustainable ROS generation. The mice were sacrificed on day 14, and excised tumors were weighed and photographed (**Figure 7C**-**D**). The weights of tumors were consistent with the relative volume of tumors. Moreover, more ROS was produced in the tumor tissue of SRG-treated mice (**Figure 7H**), which was also confirmed by the quantitative analysis (**Figure S19**). Furthermore, hematoxylin and eosin (H&E) staining of tumor tissues revealed that tumor tissues were damaged more seriously and there was more obvious apoptosis with SRG treatment compared to other treatments (**Figure 7F**), indicating that SRG had improved antitumor efficiency. After two weeks of different treatments, the weight (**Figure 7B**) and normal organs (**Figure S20**) of the mice did not change significantly, indicating that SRG had low toxicity *in vivo*. Compared to saline group, the blood biochemistry and hematology analysis of SRG group showed no obvious change (**Figure S21**), confirming the safety of SRG for antitumor therapy. Therefore, SRG had potential as a biocompatible and enhanced antitumor agent.

## Conclusion

In summary, the Mn_3_O_4_-Ce6 NPs-loaded DMSNs with HA enveloping are structured in this work as a “Sustainable ROS Generator” (SRG) for improved ROS-mediated antitumor efficiency. It is well known that the lifetime and diffusion distance of ROS are short. If ROS are only generated in partial tumor tissue, it is difficult for them to produce effective oxidative damage to the entire tumor. SRG in this study can achieve a size change by releasing small-sized MC from large-sized DMSNs under the overexpressed HAase, which helps the homogeneous distribution of Ce6 in tumor tissue and lays the foundation for homogeneous ROS generation. At the same time, Mn_3_O_4_ NPs are degraded in tumor cells, while consuming the intracellular reducing substance (GSH), which increase the intracellular ROS accumulation.

It is worth noting that PDT shows severe oxygen-dependence, while most solid tumors are hypoxia, and PDT can consume a lot of oxygen, which allows ROS to be produced in the short term, resulting in poor therapeutic effects. What's worse, a short period of oxidative stress will cause adaptive up-regulation of reducing substances in tumor cells, further weakening the effect of PDT. In this study, Mn^2+^ generated by Mn_3_O_4_ NPs in tumor cell will convert the low toxic by-product (H_2_O_2_) of PDT into the most harmful ROS (·OH) to achieve continuous production of ROS, which earns time for the oxygen recovery of tumor tissue and lays the foundation for the next phase of PDT. Such a “Sustainable ROS Generator” significantly improves ROS-mediated antitumor efficiency.

## Supplementary Material

Supplementary figures.

## Abbreviations

PDT: photodynamic therapy
PS: photosensitizer
ROS: reactive oxygen species
SRG: sustainable ROS generator
MC: Mn_3_O_4_-Ce6 nanoparticles
DMSNs: dendritic mesoporous silica nanoparticles
HA: hyaluronic acid
GSH: glutathione
·O_2_^-^: superoxide anions
H_2_O_2_: hydrogen peroxide
·OH: hydroxyl radicals;^ 1^O_2_: singlet oxygen
SOD: superoxide dismutase
EPR: enhanced permeability and retention
HAase: hyaluronidase
GSSG: oxidized glutathione
Ce6: Chlorin e6
CLSM: confocal laser scanning microscopy
3D MCSs: three-dimensional multicellular spheroids
