# Supplementary material for: Tumor cell-activated “Sustainable ROS Generator” with homogeneous intratumoral distribution property for improved anti-tumor therapy
Source: Theranostics. 2021 Jan 1;11(1):379–96. doi: 10.7150/thno.50028 (PMC7681092; doi:10.7150/thno.50028)
Supplement: Supplementary file 1 — Supplementary figures. [file thnov11p0379s1.pdf]

Supporting Information

Tumor cell-activated “Sustainable ROS Generator” with homogeneous intratumoral distribution property for improved anti-tumor therapy

Junjie Liu[1,2,3*], Xiu Zhao[1*], Weimin Nie[1], Yue Yang[1], Chengcheng Wu[1], Wei Liu[1,2,3], Kaixiang Zhang[1,2,3✉], Zhenzhong Zhang[1,2,3✉] and Jinjin Shi[1,2,3✉]

1. School of Pharmaceutical Sciences, Zhengzhou University, Zhengzhou 450001, China.
2. Key Laboratory of Targeting Therapy and Diagnosis for Critical Diseases, Henan Province, China, Zhengzhou University, Zhengzhou 450001, China.
3. Collaborative Innovation Center of New Drug Research and Safety Evaluation, Henan Province, Zhengzhou, China, Zhengzhou University, Zhengzhou 450001, China.

[*] Authors contributed equally to this work

✉ Corresponding authors: zhangkx@zzu.edu.cn; zhangzhenzhong@zzu.edu.cn; shijinyxy@zzu.edu.cn

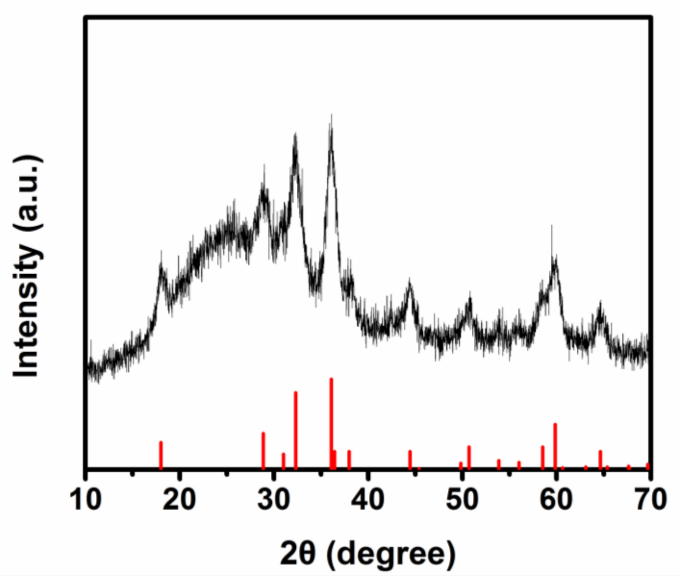

Figure S1. XRD pattern of obtained Mn_3O_4 nanoparticles (the red lines at the bottom mark the reference pattern of hausmannite Mn_3O_4 from the JCPDS database, card no.24-0734).

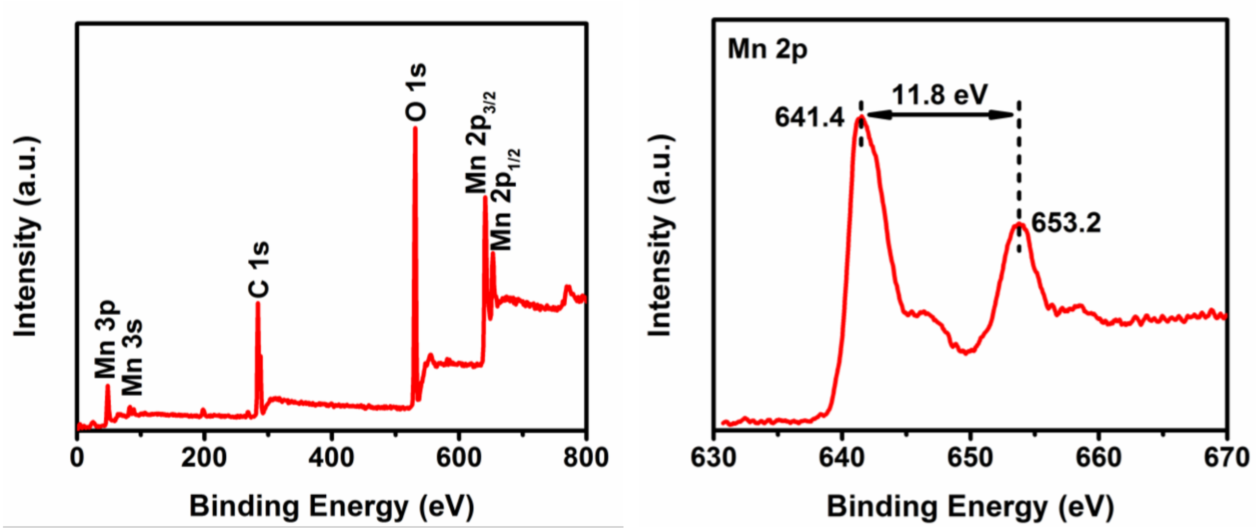

Figure S2. XPS spectrum of Mn_3O_4 and XPS spectrum of Mn 2p.

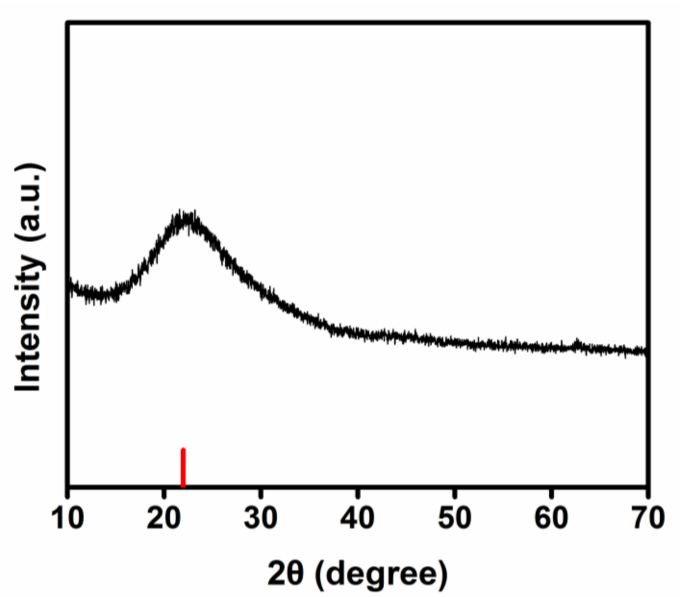

Figure S3. XRD pattern of obtained DMSN nanoparticles (the red lines at the bottom mark the reference pattern of silicon oxide from the JCPDS database, card no. 29-0085).

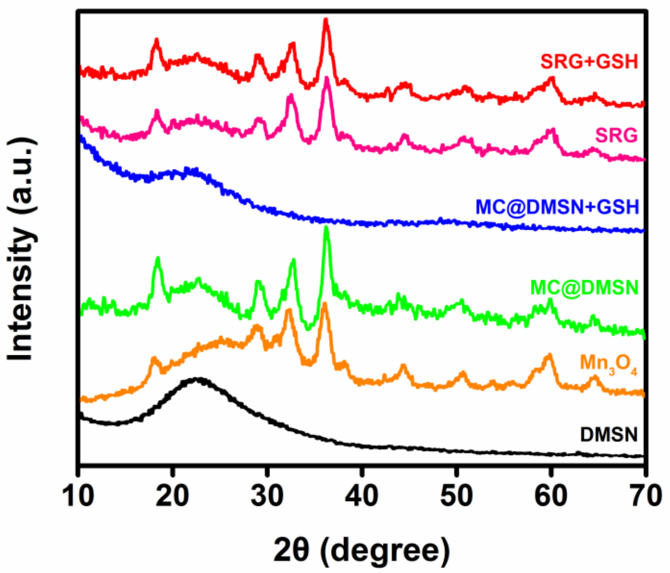

Figure S4. XRD pattern of different preparations.

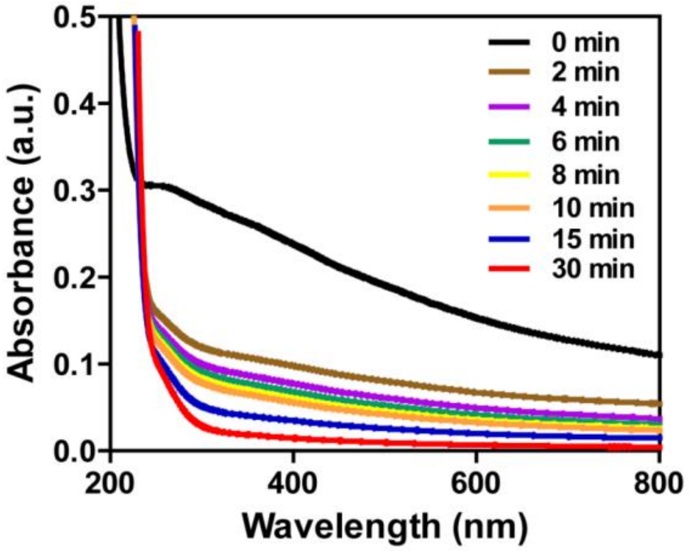

Figure S5. UV-vis absorption spectra of Mn_3O_4 treated with GSH (1 mM) for different time.

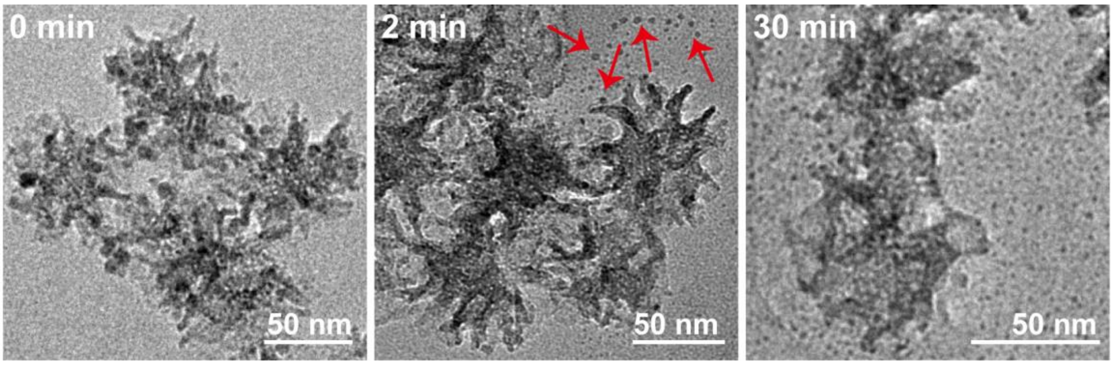

Figure S6. TEM images of MC@DMSN treated with 10 mM GSH for 0, 2 and 30 min, the red arrows represent part of un-degraded Mn_3O_4 NPs.

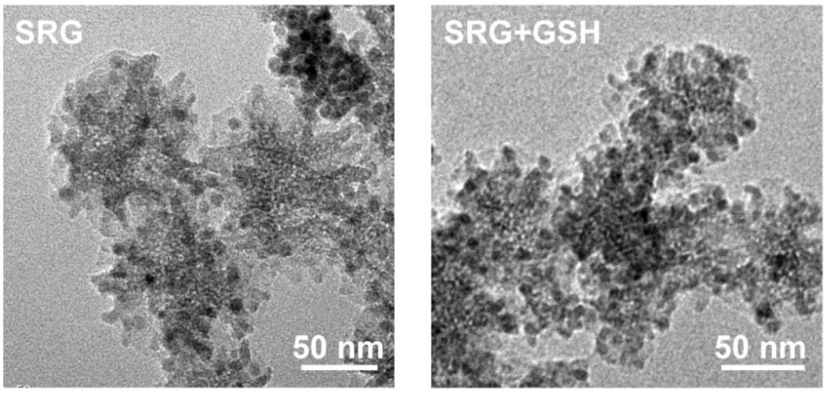

Figure S7. TEM images of SRG and SRG treated with 10 mM GSH for 30 min.

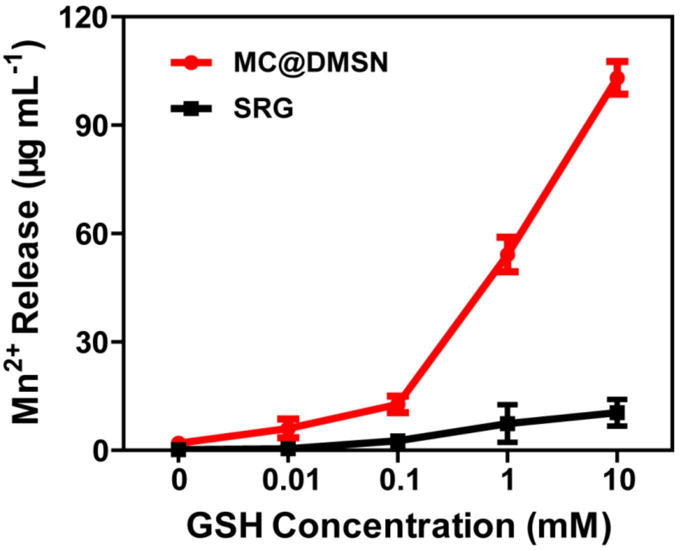

Figure S8. GSH-dependent release profiles of Mn^{2+} from MC@DMSN and SRG as measured by ICP-MS.

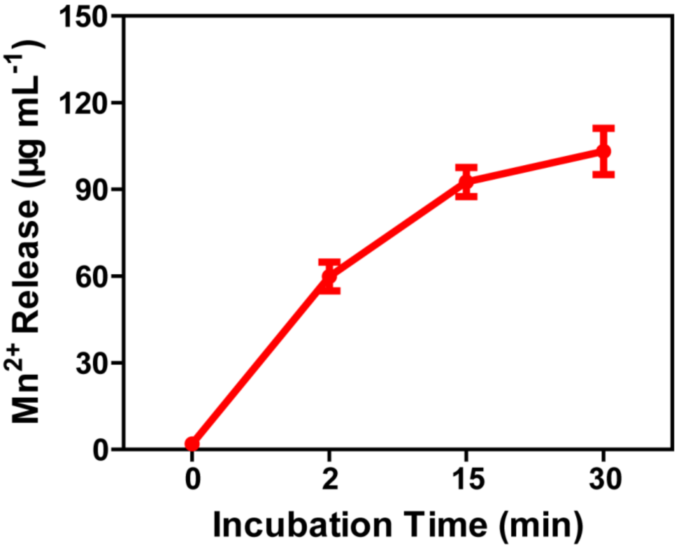

Figure S9. Time-dependent release profiles of Mn^{2+} from MC@DMSN as measured by ICP-MS.

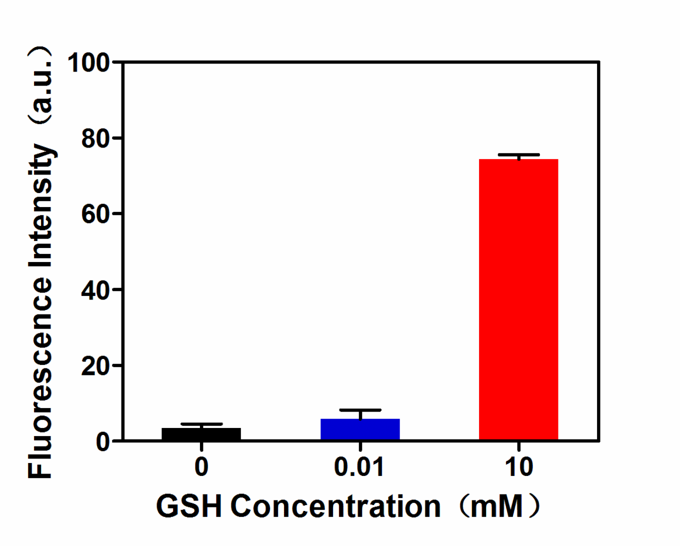

Figure S10. Response of Ce6 fluorescence intensity of MC to different concentrations of GSH (n=3).

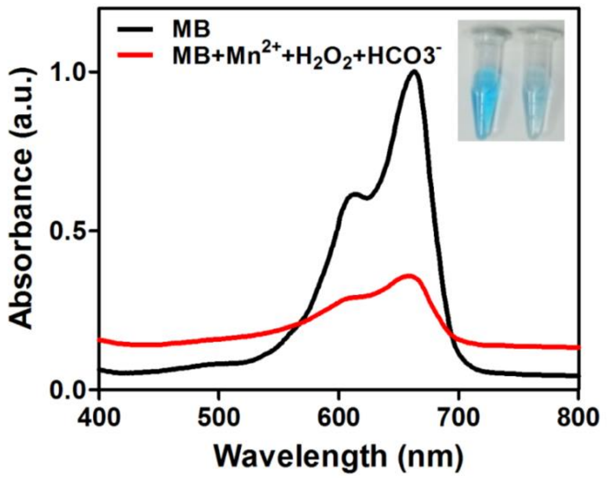

Figure S11. UV-vis absorption spectrum and photo (inset) of MB after degraded by the Mn^{2+}-mediated Fenton-like reaction.

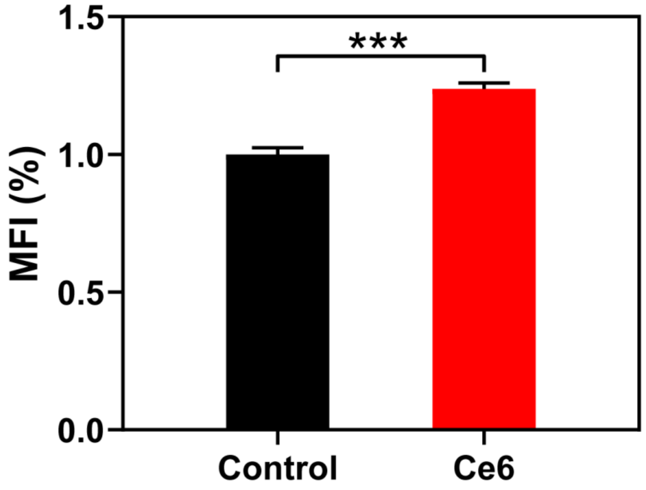

Figure S12. Mean fluorescence intensity (%) of H_2O_2 in 4T1 cells after different treatments (n=3). Results are presented as means ± s.d. *P < 0.05, **P < 0.01, and ***P < 0.001 determined by Student's t test.

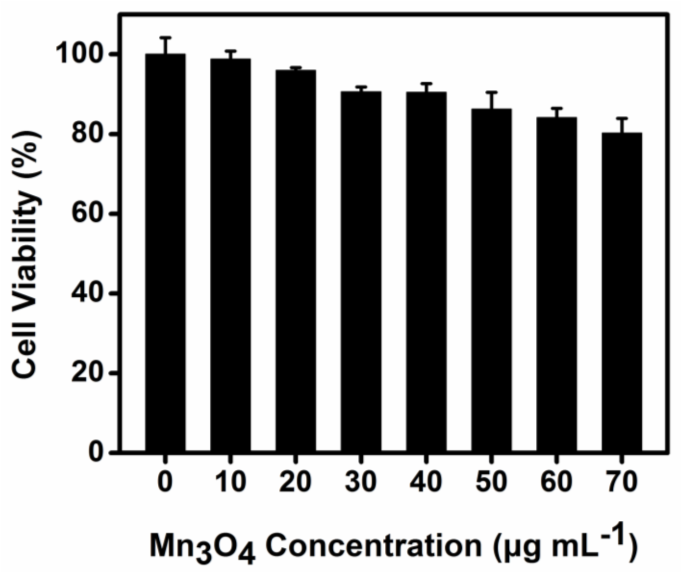

Figure S13. Cytotoxicity of Mn_3O_4 to 4T1 cells at different concentrations as indicated (n=6).

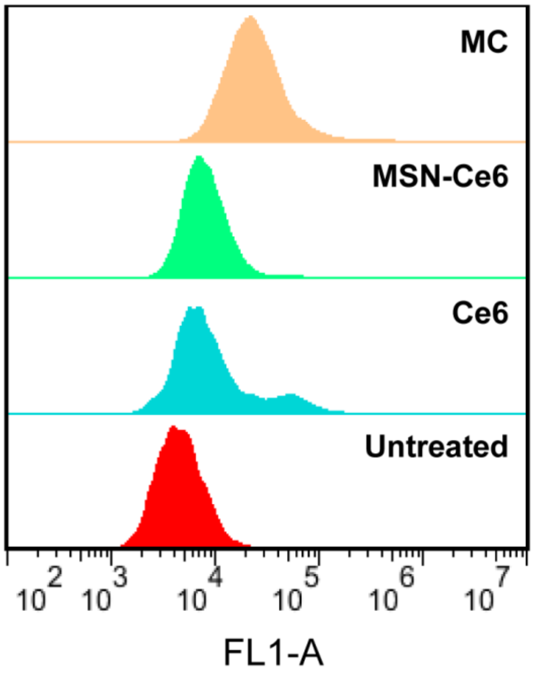

Figure S14. Flow cytometer analysis of ROS production after 4T1 cells were treated with different preparations.

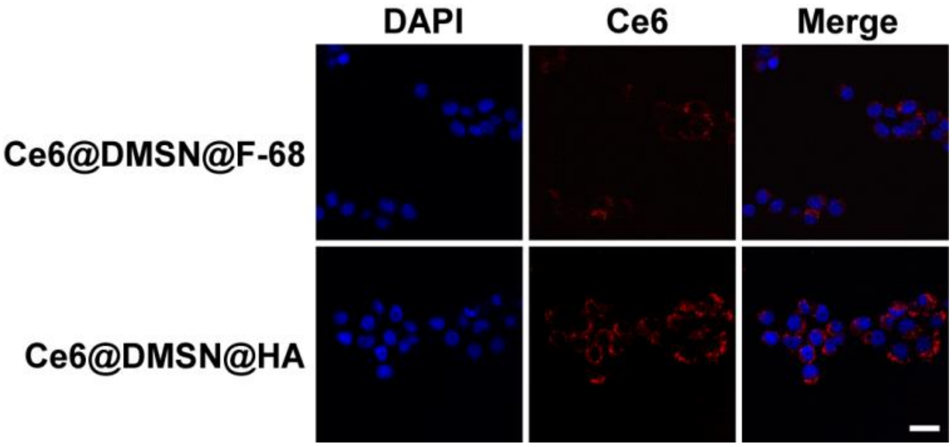

Figure S15. CLSM images of 4T1 cells incubated with Ce6@DMSN@F-68 and Ce6@DMSN@HA, respectively (scale bar: 25 μm).

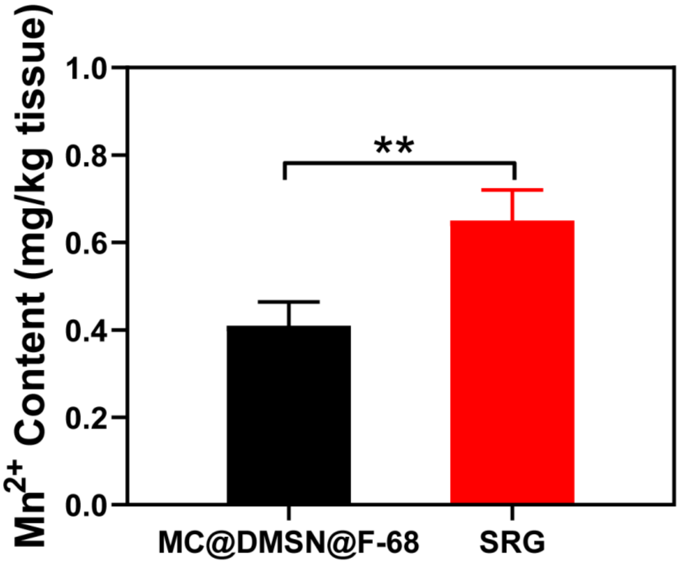

Figure S16. Mn^{2+} content in tumor tissues of tumor-bearing mice after treated with different preparations (n=3). Results are presented as means ± s.d. **P* < 0.05, ***P* < 0.01, and ****P* < 0.001 determined by Student's t test.

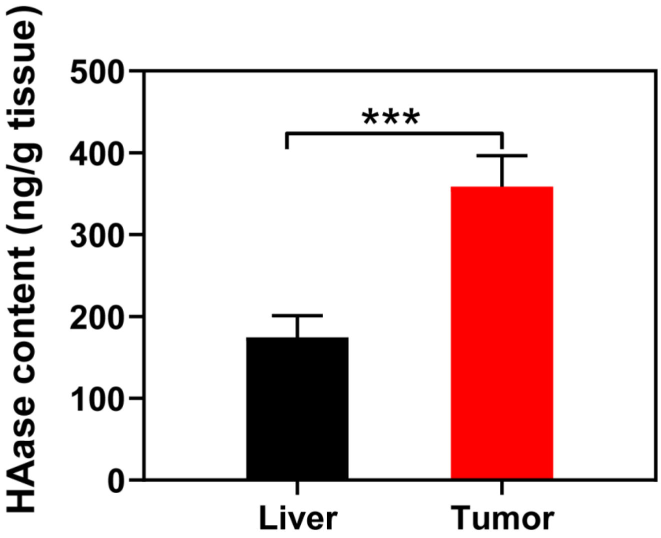

Figure S17. HAase expression in liver and tumor tissues of tumor-bearing mice (n=4)**.** Results are presented as means ± s.d. **P* < 0.05, ***P* < 0.01, and ****P* < 0.001 determined by Student's t test.

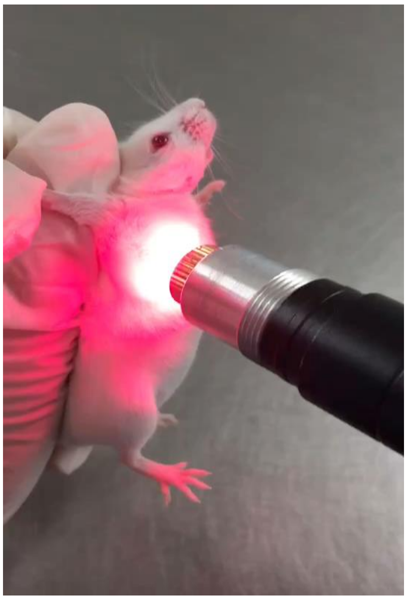

Figure S18. Images of tumor-bearing mice exposed to laser.

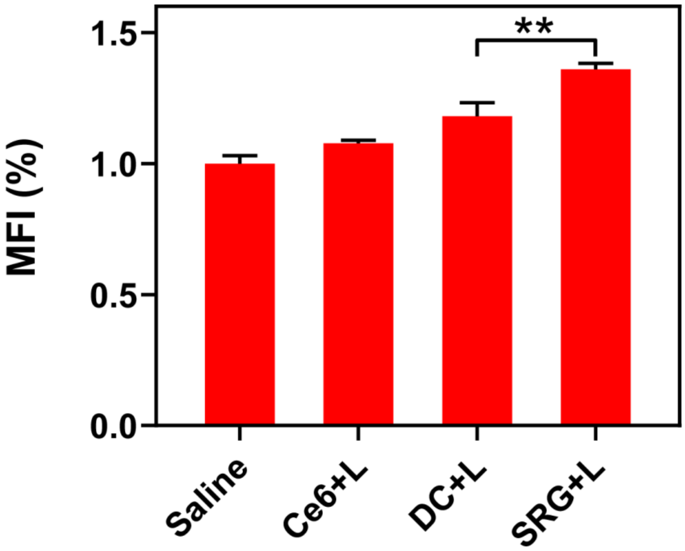

Figure S19. Mean fluorescence intensity (%) of ROS in tumor tissue (n=3). Results are presented as means ± s.d. *P < 0.05, **P < 0.01, and ***P < 0.001 determined by Student's t test.

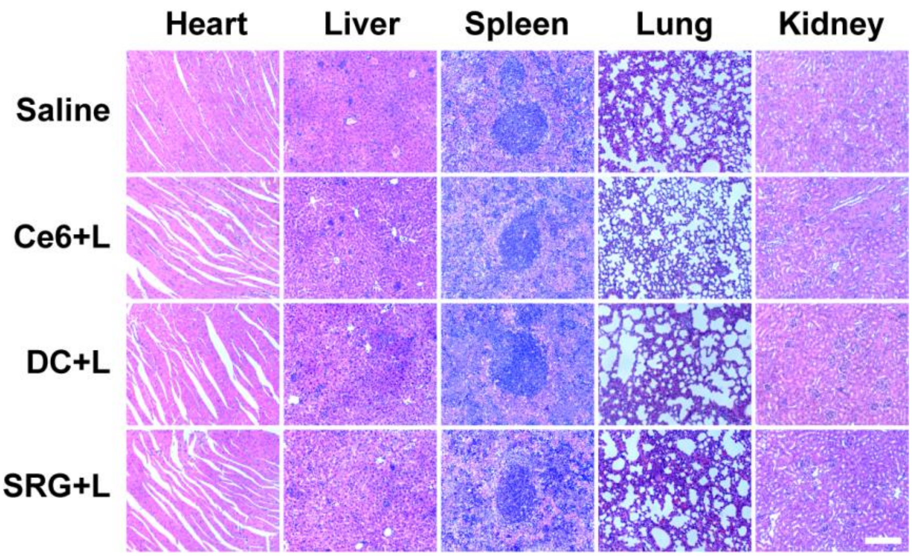

Figure S20. H&E staining of main organs from mice at 14 days post-treatment. (scale bars: 200 μm).

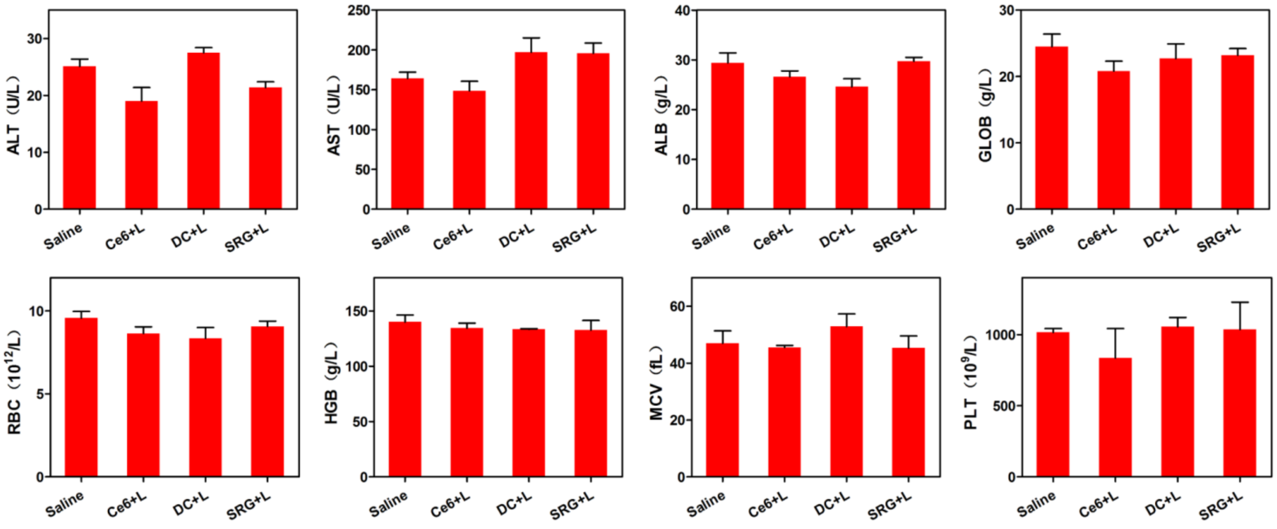

Figure S21. Blood biochemistry and hematology analysis of the mice after treatment with different preparations for 13 days.
